# Tracking Seasonal Influenza Trends in South Tyrol During 2022/2023 Using Genomic Surveillance Data

**DOI:** 10.1111/irv.70083

**Published:** 2025-03-26

**Authors:** Irene Bianconi, Mattia Manica, Elena Moroder, Giorgio Guzzetta, Stefano Merler, Piero Poletti, Elisabetta Pagani

**Affiliations:** ^1^ Laboratory of Microbiology and Virology, Provincial Hospital of Bolzano (SABES‐ASDAA) Lehrkrankenhaus der Paracelsus Medizinischen Privatuniversität Bolzano Italy; ^2^ Center for Health Emergencies Fondazione Bruno Kessler Trento Italy

**Keywords:** human influenza virus, influenza phylogenesis, phylodynamic, reproduction number, surveillance

## Abstract

**Background:**

Influenza seasons are characterized by a complex interplay of co‐circulating strains with high spatial and temporal heterogeneities. Surveillance is crucial for monitoring the spread and evolution of the virus and design effective public health response strategies.

**Aim:**

We combined epidemiological, virological, and genomic surveillance data to provide a comprehensive analysis of influenza subtypes circulating in the South Tyrol region (Italy) during season 2022/2023, leveraging phylogenetic and phylodynamic approaches.

**Methods:**

Clinical samples were collected from patients exhibiting influenza‐like symptoms and evaluated by molecular diagnostics. Whole genome sequencing was conducted, and the hemagglutinin (HA) gene sequences were used for phylogenetic analysis. A birth–death skyline model was applied to estimate strain‐specific effective reproduction numbers (Re) and attack rates.

**Results:**

Out of 4891 samples tested, 862 tested positive for influenza, of which 224 genomes were sequenced. Phylogenetic analysis of HA gene revealed A(H3N2) strains predominantly clustering in clade 3C.2a1b.2a.2b, followed by 3C.2a1b.2a.1b. A(H1N1pdm09) strains predominantly clustered in clade 6B.1A.5a.2a. Exclusive circulation of B (Victoria) subtype strains aligned with the global trend, all falling within clade V1A.3a.2. Phylogenetic analyses indicate that the strains isolated in the South Tyrol region closely resembled those circulating in the rest of Italy and Austria. Re peaked at 1.16–1.35 (95%CI) for A(H3N2), 1.06–1.34 for A(H1N1pdm09) and 1.02–1.29 for B (Victoria). 95%CI of attack rates were 6.3%–33.5% for A(H3N2), 0.6%–5.0% for A(H1N1pdm09), and 0.8%–6.5% for B (Victoria).

**Conclusion:**

Epidemiological estimates from traditional surveillance data can be corroborated by those derived from genomic sequencing, providing more robust assessments of viral transmissibility and attack rates with limited additional effort.

## 1 Background

Annual seasonal human influenza is an acute respiratory infection caused primarily by influenza A and B viruses [1]. It presents a significant global health challenge annually due to its rapid spread, potential for severe complications, and seasonal variations [1, 2, 3]. Influenza seasons are characterized by a complex interplay of co‐circulating strains with high spatial and temporal heterogeneity among regions and seasons [3, 4]. Moreover, in 2020–2022, influenza circulation has been disrupted in countries where strict containment measures against COVID‐19 were applied [5] potentially impacting the composition of circulating influenza types and subtypes [6]. The relaxation of such measures preceded a rebound of seasonal influenza, the consequence of which might have been a higher infection burden [7].

Epidemiological and virological surveillance is crucial for monitoring the spread and evolution of influenza over time [8, 9]. Surveillance systems are essential for assessing the effectiveness of interventions, such as vaccines and antiviral drugs, and for monitoring transmission [8, 10]. One crucial aspect is the selection of the strains to include in vaccines each year [11]. This process for influenza vaccines relies on systematic monitoring and analysis of prevailing influenza strains in the population. Public health authorities continuously survey the genetic and antigenic characteristics of circulating influenza viruses to anticipate and respond to the dynamic nature of viral evolution [12]. Previous studies investigated the transmission patterns and epidemiology of seasonal influenza from epidemiological [2] and/or viral genetic data [13, 14]. The systematic collection of viral genetic sequences during outbreaks can be strategic to integrate information retrieved by contact tracing investigations, provide an alert for the emergence or introduction of new variants [15, 16], and estimate key epidemiological parameters by means of phylodynamic analyses [17, 18]. The use of genomic surveillance underwent an impressive increase during the COVID‐19 pandemic, and efforts are ongoing to fully integrate them within infectious disease control programs and surveillance networks [9].

In Italy, an epidemiological surveillance system is in place to monitor influenza‐like illness (ILI) at national and regional levels, coordinated by the National Institute of Health (Istituto Superiore di Sanità, ISS) in collaboration with local public health authorities under the so‐called InfluNet and RespiVirNet network [19]. The surveillance system collects reports by sentinel general practitioners (GPs) and pediatricians as well as virological data obtained from swab tests on clinical cases. Genomic surveillance of influenza is coordinated nationally by the ISS and supported by a network of regional and provincial laboratories. Sentinel physicians collect samples, which are analyzed by reference laboratories for virus detection and genomic sequencing. Regions and provinces ensure local data collection and reporting, contributing to national and global influenza monitoring efforts.

The objective of this study was to conduct a comprehensive epidemiological and virological analysis of influenza viruses that were circulating in the Autonomous Province of Bolzano/Bozen—South Tyrol, Italy (hereafter “South Tyrol”) during the winter season of 2022/2023. South Tyrol represents less than 1% of the country's population but contributed to nearly 40% of influenza genomic samples during the 2022/2023 season in Italy, thanks to its extensive sequencing and genotyping program, fully performed by a single reference laboratory, the Laboratory of Microbiology and Virology of the Provincial Hospital of Bolzano. We investigate the application and the added value of genomic surveillance to characterize the local circulation and transmission dynamics of the influenza virus type B (Victoria) and subtypes A(H1N1)pdm09 and A(H3N2) in comparison with standard epidemiological/virological surveillance.

## 2 Methods

### 2.1 Epidemiological and Virological Surveillance

Weekly number of ILI cases notified in South Tyrol for the study period, comprised between week 46 of 2022 (November 14–20, 2022) and week 17 of 2023 (April 24–30, 2023), were obtained from the InfluNet/RespiVirNet surveillance system. The definition of ILI included cases having one of these symptoms: headache, general discomfort, and asthenia in addition to an acute onset of fever > 38°C with respiratory symptoms. The population monitored by the surveillance system ranged from 5210 to 13,087 (1.0%–2.4% of the total province population), depending on the weekly adherence of the sentinel doctors.

In this study, the criteria for conducting nasopharyngeal swabs were defined based on the presence of ILI symptoms identified by clinicians in patients seeking hospital care. Due to limitations in accessing detailed clinical data, the collected variables were restricted to the patient's date of birth and the entity requesting the analysis, which included various hospital departments and outpatient clinics (Table S2).

Naso‐pharyngeal swabs collected in South Tyrol under InfluNet/RespiVirNet for the 2022/2023 season underwent multiplex real‐time PCR assays designed to detect not only influenza viruses but also other respiratory viruses, following the network's operational protocol. The following kits were used during routine analyses: Allplex Respiratory Full Panel (Arrow Diagnostics, South Korea); BIOFIRE Respiratory 2.1 plus Panel e Pneumonia Panel plus (bioMérieux, France); Xpert Xpress CoV‐2/Flu/RSV plus (Cepheid, USA).

### 2.2 RNA Extraction and Genome Sequencing

Samples were sequenced by the Laboratory of Microbiology and Virology at the Provincial Hospital of Bolzano, representing the only reference laboratory in South Tyrol for genomic surveillance of influenza in human samples. All strains belonged to a different and unique patient. Influenza virus genomic sequencing was conducted on all samples with Ct values ≤25 in real‐time PCR. This threshold ensures that only samples with sufficient viral genetic material are included for sequencing, maximizing the chances of obtaining high‐quality genetic data. To maintain the integrity and reliability of the dataset, we excluded sequences displaying suboptimal quality, including those with undetermined (N) bases within the genetic code or frameshift mutations. A subset of positive samples underwent viral RNA extraction using the MagMAX Viral/Pathogen Nucleic Acid Isolation Kit (ThermoFisher Scientific, USA) on the KingFisher Flex system (ThermoFisher Scientific, USA) following the manufacturer's protocol. Genomic library preparation procedures were conducted using the NGS STAR system (Hamilton Company, USA). Library preparation involved the utilization of the RNA Prep with Enrichment kit and the Respiratory Virus Oligo Panel v2 kit (Illumina, USA), following manufacturer's protocols. Libraries were run on the MiSeq platform (Illumina, USA) (sequencing steps are summarized in Figure S7). Read assembly and genome analysis were performed using the BASESPACE online server (Illumina, https://basespace.illumina.com). Specifically, Explify RPIP Data Analysis 2.1.1 and DRAGEN RNA Pathogen Detection 3.5.17 were used. The haemagglutinin (HA) gene sequences of the samples meeting quality criteria were submitted to GISAID (Global Initiative on Sharing All Influenza Data, https://gisaid.org/) [20].

### 2.3 Phylogenetic Analysis

We included all sequences from samples collected in South Tyrol, reference strains recommended by the National Influenza Center of the Istituto Superiore di Sanità, which serves as the National Influenza Centre (NIC), as well as vaccine strains for the 2022/2023 season for comparative purposes. To enrich the phylogenetic analysis and consider the viral circulation in the neighboring areas, selected sequences from Italy and Austria (which borders South Tyrol) freely available on GISAID were also included, with a focus on strains harboring complete HA genes. The HA gene sequences of influenza strains were aligned with the BioEdit software employing the ClustalW Multiple alignment algorithm. The MEGA11 software was employed for phylogenetic tree generation. Phylogenetic analysis was conducted using the Maximum Likelihood (ML) method with a Tamura–Nei substitution model. As a sensitivity analysis, we additionally used a neighbor‐joining (NJ) method with a maximum composite likelihood substitution model. Bootstrap testing comprising 1000 replicates was adopted to assess the reliability of branch grouping. To enhance the readability of figures, phylogenetic trees, reported in Figures S1–S6, are presented by showing only one representative strain for each unique HA sequence.

### 2.4 Phylodynamic Analysis

We performed a Bayesian phylodynamic analysis using a Markov Chain Monte Carlo approach (MCMC) applied to each subtype (B, A(H1N1)pdm09, A(H3N2)) separately. A birth–death skyline model (BDSKY) [21] was used to estimate temporal changes in the reproductive number (Re) associated with each influenza subtype, the proportion of collected samples over the total infections in the population (“sampling proportion”), the duration of the infectious period, and the time to the most recent common ancestor (tMRCA). Leveraging the estimated values of the sampling proportions, we derived estimates of subtype‐specific attack rates. The model assumes a general time reversible (GTR) + G nucleotide substitution model and a strict molecular clock following a lognormal prior distribution of mean 0.002 nucleotide substitutions per site per year, implying that every branch in a phylogenetic tree evolves according to the same evolutionary rate. To investigate changes in transmissibility during the study period, we assumed each subtype specific Re to be piecewise constant. We run a sensitivity analysis where we re‐estimated the transmissibility after assuming a fixed tree topology as estimated from the phylogenetic analysis, and another sensitivity analysis where we used a model with an uncorrelated, lognormally distributed, relaxed molecular clock. We carried out 50 million independent MCMC runs for each analysis, sampling every 5000 steps with a 10% burn‐in. We used the BEAST2 software (v2.7.5) for phylodynamic analysis and the software TRACER (v.1.7.2) to inspect the parameter posterior distributions and assess convergence and sufficient sampling (effective sample size >200). Additional information on model parametrization, choice of priors, and sensitivity analyses are available in the Supplementary Text.

### 2.5 Estimates of Influenza Transmission From Epidemiological and Virological Surveillance Data

We estimated the exponential growth rate of each influenza subtype from the time series of the weekly incidence, obtained by multiplying the total reported ILI incidence (number of reported ILI cases divided by the number of patients served by sentinel doctors) by the relative abundance of each subtype, as identified by molecular diagnosis in nasopharyngeal swabs from the same week. We then estimated the subtype‐specific reproduction numbers R using the equation R=1+rG, where *r* represents the subtype‐specific exponential growth rate and *G* represents the average generation time, assumed to be 2.7 days [2, 22, 23].

## 3 Results

### 3.1 Surveillance of Influenza in South Tyrol, 2022/2023

**FIGURE 1:**
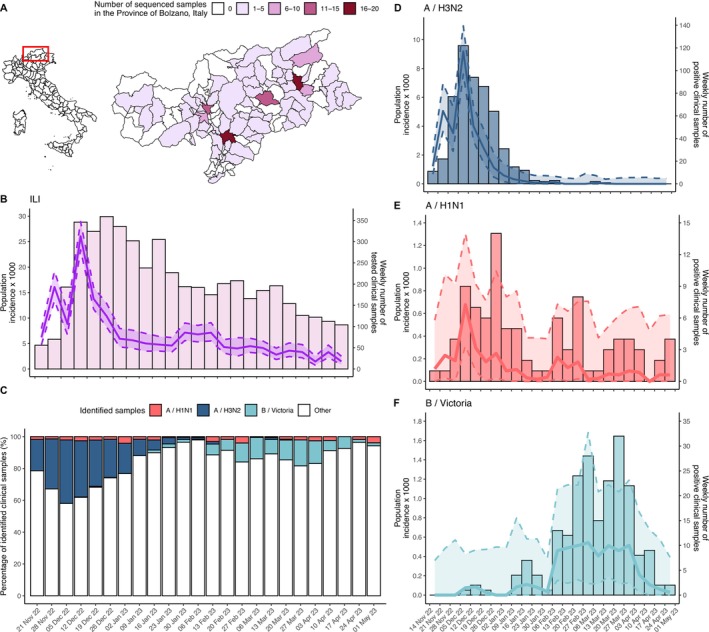
(A) Number of sequenced clinical samples by municipality of residence in South Tyrol, Italy. (B) Estimated influenza‐like illness (ILI) incidence in the general population (solid lines), its 95% confidence intervals (shaded area within dashed lines), and weekly number of tested clinical samples (bars). (C) Results of PCR testing for influenza subtypes by week. (D) Estimated incidence of A(H3N2) subtype in the general population (solid lines) and weekly number of A(H3N2) positive samples (bars). (E) Estimated incidence of A(H1N1)pdm09 subtype in the general population (solid lines) and weekly number of A(H1N1)pdm09 positive samples (bars). (F) Estimated incidence of B (Victoria) subtype in the general population (solid lines) and weekly number of B (Victoria) positive samples (bars).

Between November 14, 2022, and April 30, 2023, 1576 ILI cases were reported in South Tyrol, Italy, in the InfluNet/RespiVirNet surveillance system. A total of 4891 nasopharingeal swabs were tested for flu‐like symptoms, 862 (17.6%) of which tested positive for influenza viruses. A total of 184 of the positive samples (21.3%) were from individuals aged 0–4 years, 152 (17.6%) were from the 5–14 age bracket, 240 (27.8%) were from the 15–64 demographic, and 286 (33.2%) were from individuals aged 65 years or older. Among the positive cases, 547 (63.4%) were identified as A(H3N2) subtype, 93 (10.8%) were A(H1N1)pdm09, and 210 (24.4%) B (Victoria) (Table 1). In addition, 12 samples (1.4%) tested positive for influenza type A, but the specific subtype could not be determined due to incomplete test results, which were intrinsic to the sample, such as insufficient material, improper storage, and other factors independent of the testing center. No cases of co‐infections were recorded. A total of 224 positive samples were fully sequenced, of which 155 were A(H3N2) lineages (September 1, 2022, to March 14, 2023), 29 were A(H1N1)pdm09 (October 14, 2022, to March 27, 2023), and 40 were B (Victoria) (January 9 to April 11, 2023). The sequenced samples came from individuals living within 67 of the 116 municipalities (58%) of the province (Figure 1A), which make up 81.9% of the total population. The metadata for all South Tyrol sequences, including patient age (grouped into 10‐year intervals, e.g., 0–9, 10–19, …) and residence, aggregated by health district, are provided in Table S2.

**TABLE 1:** Positive samples for influenza viruses by real‐time PCR per week of surveillance during the 2022/2023 season. The table shows the total number of samples tested for respiratory viruses and the number of samples resulted positive for influenza per week of surveillance. The data was obtained from the RespiVirNet platform (https://www.epicentro.iss.it/influenza/respivirnet).

Surveillance week	Total tested samples	A(H3N2)	A(H1N1)pdm09	A not subtyped	B (Victoria)
2022‐46	56	11	1	0	0
2022‐47	70	22	1	0	0
2022‐48	193	78	4	0	0
2022‐49	346	123	9	1	1
2022‐50	324	94	7	0	2
2022‐51	359	86	6	2	1
2022‐52	336	64	14	2	0
2023‐1	302	31	5	3	0
2023‐2	238	15	5	0	4
2023‐3	305	12	2	0	7
2023‐4	227	3	1	0	4
2023‐5	194	2	1	0	1
2023‐6	192	3	6	0	13
2023‐7	175	0	3	0	12
2023‐8	201	0	8	0	24
2023‐9	208	0	1	0	28
2023‐10	166	2	1	0	15
2023‐11	185	1	4	1	23
2023‐12	196	0	3	1	32
2023‐13	154	0	4	0	22
2023‐14	126	0	1	2	8
2023‐15	122	0	0	0	9
2023‐16	112	0	2	0	2
2023‐17	104	0	4	0	2
Total	4891	547	93	12	210

### 3.2 Temporal Patterns in the Circulation of Influenza Sub‐Types in South Tyrol, 2022/2023

The peak of the influenza epidemic was reached during surveillance week 49/2022 (December 5 to 11, 2022; Figure 1B). At the beginning of the season, the A(H3N2) subtype was dominant, with only a small proportion of A(H1N1)pdm09 samples (Figure 1C). After a peak of 9.19 cases per 1000 population in week 49/2022, the incidence of influenza A(H3N2) gradually decreased, with the last positive sample of this subtype recorded in week 11/2023 (Figure 1D). Samples positive for A(H1N1)pdm09 were detected throughout the influenza season, with the peak of incidence occurring in the same week as A(H3N2) at 0.68 cases per 1000 population (Figure 1E). The influenza B (Victoria) lineage was first identified in week 49/2022 and remained at a low level until week 2/2023, after which it gradually increased, becoming dominant during week 6/2023 with an incidence hovering around 0.54 cases per 1000 between February 6 and April 3, 2023 (Figure 1F).

**FIGURE 2:**
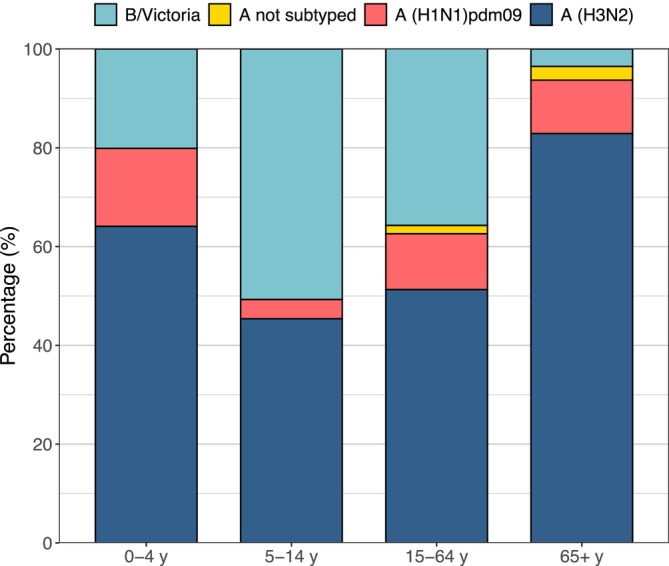
Influenza subtype positivity by age. Relative abundance of samples positive for influenza viruses by subtype by age group (0–4 years, 5–14 years, 15–64 years, and 65 years or older).

The dataset containing positive samples of influenza virus was stratified by age cohorts. The A(H3N2) subtype dominated in all age groups except the 5–14 cohort, with a relative abundance of 64.1% in the 0–4 age group, 51.3% in the 15–64 age group, and 82.9% in those 65 years and older (Figure 2). On the other hand, the 5–14 age group showed a higher frequency of influenza virus type B (50.7%) compared with sporadic isolation in the elderly cohort (3.5%). In the 0–4 and 15–64 age groups, influenza virus type B accounts for 20.1% and 35.8% of all samples, respectively. The A(H1N1)pdm09 subtype was present in all age groups, although with lower frequencies (15.8% in the 0–4 age group, 3.9% in the 5–14 age group, 11.3% in the 15–64 age bracket, and 10.8% among those over 65).

### 3.3 Circulating Influenza Clades in South Tyrol, 2022/2023

The phylogenetic analysis revealed two main clusters (A and B) of influenza A(H3N2) viruses, as shown in Figure S1. The majority of influenza A(H3N2) strains from South Tyrol (111 out of 151) were clearly clustered within sub‐clade 3C.2a1b.2a.2b. The second most prevalent sub‐clade is 3C.2a1b.2a.1b (35 out of 151), followed by 3C.2a1b.2a.3a.1, which includes only two strains, both originating from patients residing in the same town. Three sub‐clades contained only one strain each. Notably, strains from Austria and other Italian regions were also classified within both clusters (A and B). Analysis of the phylogenetic tree clusters did not reveal a clear correlation between the isolation date of the strain and its position within the tree. Strains isolated during both the early (2022) and late (2023) influenza season were distributed across the main clusters. Furthermore, the short branch lengths of strains isolated in South Tyrol indicated their genetic similarity to one another.

Most of influenza A(H1N1)pdm09 strains (25 out of 27) circulating in South Tyrol belonged to clade 6B.1A.5a.2a (Figure S2). Two additional strains, isolated in other Italian regions, were also classified within this same clade. Two strains from South Tyrol clustered together with three Austrian strains, one strain from the province of Milan, and the A/Wisconsin/67/2022 strain, which was included in the vaccine composition for the 2023/2024 season. All these South Tyrol strains were identified within sub‐clade 6B.1A.5a.2a.1. Notably, one strain from South Tyrol (A/Bolzano/7/2023) exhibited a high similarity to the A/Wisconsin/67/2022 vaccine strain, as evidenced by their shared branch origin and relatively short branch lengths.

Only strains belonging to the B Victoria lineage were identified in South Tyrol in season 2022/2023. All circulating strains belonged to the V1A.3a.2 sub‐clade (Figure S3). The strains isolated in South Tyrol are very similar to those that circulated in the rest of Italy and Austria. The strains are categorized into three main clusters: cluster A, which comprises 23 strains; cluster B, which comprises eight strains; and cluster C, which comprises a single strain.

The clustering of isolates within circulating clades remained robust when using the NJ approach for the phylogenetic analysis instead of the ML one (see Figures S4–S6).

### 3.4 Epidemiological Estimates for Influenza Sub‐Types in South Tyrol, 2022/2023

**FIGURE 3:**
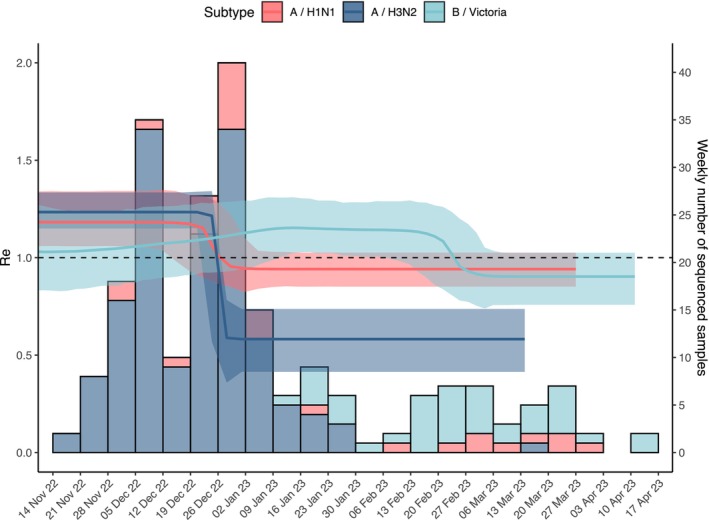
Weekly number of sequenced clinical samples (bars) and estimated transmissibility (Re) for A(H3N2), A(H1N1)pdm, and B (Victoria) (lines: mean; shaded areas: 95% highest posterior density interval).

According to the phylodynamic model, Re remained above 1 from the start of the study period until the end of December 2022 for A subtypes and from early January 2023 until the second half of February 2023 for B (Victoria) (Figure 3). The value of Re at the peak, estimated with the phylodynamic model, was generally consistent with lineage‐specific reproduction numbers obtained from epidemiological surveillance data (i.e., by estimating the exponential growth of the incidence curves; see Table 2). In particular, the peak Re values were estimated at 1.24 (95%CrI 1.16–1.35) for A(H3N2), 1.19 (95%CrI 1.06–1.34) for A(H1N1)pdm09, and 1.15 (95%CrI 1.02–1.29) for B (Victoria) when using the phylodynamic model. By comparison, corresponding estimates from the exponential growth method were 1.33 (95%CrI 1.03–1.64) for A(H3N2), 1.29 (95%CrI 1.14–1.44) for A(H1N1)pdm09, and 1.14 (95%CrI 0.98–1.29) for B (Victoria). Differences by subtype and estimation method were not significant, as shown by the broadly overlapping credible intervals.

**TABLE 2:** Estimated mean and 95% confidence intervals for the reproduction number from influenza‐like infections (ILI) and genomic surveillance, length of infections, and sequence sampling proportion.

Influenza subtype	Peak Re from phylodynamic	R from incidence curves	Duration of infectious period (days)	Sampling proportion
A(H3N2)	1.24 (1.16–1.35)	1.33 (1.03–1.64)	4.05 (2.89–5.48)	0.23% (0.09–0.47%)
A(H1N1)pdm09	1.19 (1.06–1.34)	1.29 (1.14–1.44)	4.07 (2.79–5.76)	0.39% (0.11–0.98%)
B (Victoria)	1.15 (1.02–1.29)	1.14 (0.98–1.29)	4.07 (2.8–5.71)	0.39% (0.12–0.91%)

The phylodynamic model also yielded similar estimated durations of infectiousness across influenza subtypes, ranging between 2.8 and 5.8 days, consistently with available estimates for the influenza generation time (~2.7 days [21]). The estimated sampling proportion was comprised between 0.1% and 1% for all subtypes, resulting in an estimated attack rate in the general population of about 6.3%–33.5% for A(H3N2), 0.6%–5% for A(H1N1)pdm09, and 0.8%–6.5% B (Victoria). The time to the most recent common ancestor (tMRCA) was estimated to fall between January 28 and March 2, 2022 for A(H3N2) (95% highest posterior density interval), between February 5 and May 11, 2022, for A(H1N1)pdm09 and between June 13 and October 10, 2022, for B (Victoria). Sensitivity analysis considering either an optimized relaxed molecular clock or a fixed tree topology yielded comparable results (see Table S1 in the Supplementary Text).

## 4 Conclusions

Influenza viruses pose a significant global public health challenge, resulting in substantial morbidity, mortality, and economic burden each year [24]. Understanding the dynamics of influenza transmission, including the circulation of viral strains, their transmission potential, and the effectiveness of vaccination campaigns, is crucial for informing public health interventions and mitigating the impact of influenza epidemics. This study improves the surveillance of influenza viruses circulating during the 2022/2023 influenza season in South Tyrol, Italy, by combining epidemiological, virological and genomic data.

The circulation of seasonal influenza in South Tyrol in 2022/2023 exhibited an early onset, similarly to pre‐COVID‐19 periods [25], with a peak incidence occurring in early December 2022 (week 49/2022). A pronounced decrease in the number of samples testing positive for influenza viruses was observed from the end of December. This decline marked the conclusion of the peak activity period for influenza subtype A(H3N2), which had dominated the early phase of the season. Conversely, the activity of A(H1N1)pdm09 remained limited throughout the surveillance period. The activity of influenza virus type B began only after the decline of the influenza A wave. This clear temporal separation of influenza types is a common pattern in Italy: In a study analyzing 13 winter seasons between 2004 and 2017, influenza A and B had overlapping activities in only three seasons, and type B viruses circulated 4 weeks later than type A viruses on average [4]. The peak of type A activity at the end of 2022 and that of type B in February/March 2023 in South Tyrol mirrored the temporal dynamics observed in European countries, including Italy and Austria [26], and in the United States [27]. The reasons for this temporal separation of subtype activity are poorly understood and are likely the result of complex ecological dynamics among viral subtypes, mediated by cross‐immunity effects and by the immune escape driven by antigenic mutations [28]. Several factors may be at play, such as the competition for the infection of susceptible individuals, the combination of differences in age‐specific susceptibility profiles across subtypes [2] with social activities over time (e.g. school and holiday calendars), and temporal heterogeneities in the reintroduction of type‐specific strains from other countries at the beginning of each season. Further research is needed to clarify the determinants of temporal distributions of seasonal influenza subtype activity. The dominance of A(H3N2) identified in South Tyrol data in 2022/2023 reflects analogous results recorded internationally [29]; the earlier onset and peak of heightened influenza activity had been anticipated by observations from the Southern Hemisphere [30].

A main added value of this study consisted in phylogenetic and phylodynamic analysis of 224 full genome sequences, which represented about 37% of total influenza human strains genotyped in Italy throughout the period (39.9% and 27.8% of total influenza A and B, respectively).

Phylogenetic analysis of A(H3N2) influenza viruses showed the presence of two primary genetic clusters among South Tyrolean samples: sub‐clade 3C.2a1b.2a.2b and sub‐clade 3C.2a1b.2a.2a.1b. The majority of strains from South Tyrol mainly clustered within the former, the same clade that was prevalent in the previous influenza season too [25]. Comparison of the genetic sequences of these circulating strains with those of the vaccine strains included in the 2022/2023 influenza vaccine formulation revealed a notable degree of genetic similarity, suggesting that the strains circulating in the South Tyrolean population shared key genetic characteristics with the vaccine strains, potentially enhancing the effectiveness of the vaccine. For A(H1N1)pdm09 viruses, most of the sequenced strains from South Tyrol were found to be part of sub‐clade 6B.1A.5a.2a. We found notable antigenic differences between the circulating strains of A(H1N1)pdm09 in South Tyrol and the vaccine strains recommended for the 2022/2023 season. This result aligns with previous findings from the World Health Organization on the misalignment of circulating and vaccine strains for A(H1N1)pdm09 [31], which had prompted adjustments to the vaccine compositions for the subsequent season (2023/2024) in the northern hemisphere to enhance efficacy against evolving viral strains. The only lineage of circulating influenza B viruses was Victoria, specifically sub‐clade V1A.3a.2, in line with the probable extinction of the B (Yamagata) lineage suggested in other studies [32]. The circulating strains showed antigenic similarity to the vaccine strain B/Austria/1359417/2021, supporting its continued use in future seasons.

By applying a phylodynamic model on genomic surveillance data, we estimated peak reproduction numbers ranging between 1 and 1.35, with a timing of the lineage‐specific peaks that was consistent with the observed switch of circulation between A and B lineages. Furthermore, these estimates were well aligned with those obtained from traditional epidemiological surveillance data. The phylodynamic models also suggested that 6.3%–33.5% of the population of South Tyrol might have been infected with A(H3N2), 0.6%–5% with A(H1N1)pdm09, and 0.8%–6.5% with B (Victoria) during the 2022/2023 season. These estimates are within the range of the subtype‐specific attack rates estimated at the national level in pre‐pandemic periods using different methods [2]. The estimation of subtype specific attack rates in the population over multiple seasons, combined with longitudinal data on vaccine uptake and composition, may provide insights on ecological mechanisms causing the relative abundance of different subtypes, thereby supporting strategies for vaccine design. Other estimates provided by the phylodynamic analysis of available sequences were consistent with those available in literature. For instance, estimates of the duration of the infectious period (ranging between 2.8 and 5.7 days for the circulating strains during the 2022/2023 influenza season) are in agreement with previously reported values between 2 and 5 days [33] and with reports of undetectable viral shedding 6–7 days after onset of clinical illness for influenza A virus [1].

Surveillance data have intrinsic limitations related to sampling and data collection. The population coverage of sentinel doctors in the epidemiological surveillance network was limited (1%–2.4% of the total population), potentially biasing ILI incidence estimates; this resulted in broad confidence intervals for the estimated incidence of lineages A(H1N1)pdm09 and B (Victoria). Virological analysis was based on clinical samples, under the assumption that the population of hospital patients is representative of the general population. Genomic surveillance may be also affected by significant sampling bias due to smaller sizes; on the other hand, the stringent quality criteria adopted ensure the accuracy of strain relationship reconstruction in the phylogenetic and phylodynamic analyses. The unavailability of patient clinical and vaccination data precluded considerations of relative viral pathogenicity and vaccine effectiveness. In the absence of data on antigenic and phenotypic traits, characteristics of the strains were solely discussed based on genomic analyses, limiting the extent of interpretation within the study. These limitations apply also to the statistical analyses.

Although all sequences available from the province were included in our analysis, the phylogenetic tree reconstruction is subject to limitations, including potential sampling bias from the additional strains selected in the rest of Italy and Austria, which may not fully represent the entire viral population. Nonetheless, we believe that our conclusions on circulating clades in South Tyrol in 2022/2023 are not significantly affected by potential sampling biases. Our analysis does not provide information on the circulation of influenza at a broader spatiotemporal scale.

The phylogenetic tree estimation method can influence the reliability of inferred evolutionary relationships. While the ML method is considered more accurate, it is computationally intensive and the NJ method may be preferred for its efficiency in handling large datasets and its ability to produce reliable results in epidemiological surveillance [34]. Our analysis showed consistent clustering of isolates within circulating clades when using the ML (Figures S1–S3) and the NJ method (Figures S4–S6). The adopted birth–death phylodynamic model requires strong informative priors to accurately estimate the parameters [35]. Furthermore, it assumes that sampled individual in the trees do not transmit infection after sampling; in our case, this assumption should be appropriate given that samples were obtained from hospitalized patients who are isolated upon diagnosis.

Despite the above limitations, results from our analyses were consistent with epidemiological trends observed in Italy and neighboring Austria, and internationally; furthermore, quantitative estimates from the phylodynamic model were validated by traditional methods (e.g., for what concerns subtype‐specific reproduction numbers) and the available literature (duration of infectious period, estimated attack rates), demonstrating the overall robustness of results.

Our study shows the added value of integrating routine epidemiological and virological surveillance with genomic surveillance for the identification and quantification of trends in influenza transmission dynamics. Useful insights from genetic analyses include information on vaccine strain selection processes and potential vaccine effectiveness against circulating strains. The quantitative estimation of parameters such as reproduction numbers and subtype‐specific attack rates corroborates those obtained with traditional methods, allowing for more robust epidemiological assessments. The future scale‐up of genomic surveillance might facilitate the identification of interregional differences in influenza subtype circulation, helping to shed light on their complex ecological dynamics.

## Author Contributions

Conceptualization: I.B., S.M., P.P., and E.P.; methodology: I.B., M.M., E.M., P.P., S.M., and E.P.; software: I.B. and M.M.; validation: I.B., S.M., P.P., and E.P.; formal analysis: I.B., M.M., and E.M.; investigation: I.B., M.M., E.M., P.P., S.M., and E.P.; resources: S.M. and E.P.; data curation: I.B., E.M., and E.P.; writing – original draft preparation: I.B., M.M., E.M., and G.G.; writing – review and editing: I.B., M.M., E.M., P.P., G.G., S.M., P.P., and E.P.; visualization: I.B., M.M., and E.M.; supervision: I.B, S.M., P.P., and E.P. All authors have read and agreed to the published version of the manuscript.

## Ethics Statement

This study was approved by the Ethics Committee for Clinical Trials of the Autonomous Province of Bolzano at its meeting on 18/10/2023 (Opinion No. 96‐2023).

## Consent

The data presented in this study were collected as part of a routine surveillance activity coordinated by the Italian National Institute of Health (Istituto Superiore di Sanità, ISS). As such, the requirement for informed consent for publication does not apply.

## Conflicts of Interest

The authors declare no conflicts of interest.

### Peer Review

The peer review history for this article is available at https://www.webofscience.com/api/gateway/wos/peer‐review/10.1111/irv.70083.

## Permission to Reproduce Material From Other Sources

Not applicable.

## Supporting information


**Table S1.** Estimated mean and 95% confidence intervals for the reproduction number from genomic surveillance, length of infections, sequence sampling proportion, and time to most recent common ancestor according to different prior choices for the molecular clock and tree topology.
**Figure S7.** Sequential steps of the analyses. The number of samples at each step is reported in the boxes.

**Table S2.** Metadata related to the isolates of the influenza viruses analyzed.

**Data S1.** Supplementary information.

**Figure S1.** Phylogenetic relationships of the HA gene of influenza viruses isolated in South Tyrol as obtained for A(H3N2) using the maximal likelihood (ML) method with a Tamura–Nei substitution; red and pink: vaccine strains; blue: strains from Italy; green: strains from Austria; bold: reference strains. Trees are midpoint‐rooted.

**Figure S2.** Phylogenetic relationships of the HA gene of influenza viruses isolated in South Tyrol as obtained for A(H1N1)pdm09 using the maximal likelihood (ML) method with a Tamura–Nei substitution; red and pink: vaccine strains; blue: strains from Italy; green: strains from Austria; bold: reference strains. Trees are midpoint‐rooted.

**Figure S3.** Phylogenetic relationships of the HA gene of influenza viruses isolated in South Tyrol as obtained for B (Victoria) using the maximal likelihood (ML) method with a Tamura–Nei substitution; red and pink: vaccine strains; blue: strains from Italy; green: strains from Austria; bold: reference strains. Trees are midpoint‐rooted.

**Figure S4.** Phylogenetic relationships of the HA gene of influenza viruses isolated in South Tyrol as obtained for A(H3N2) using the neighbor‐joining method with a maximum composite likelihood substitution model; red and pink: vaccine strains; blue: strains from Italy; green: strains from Austria; bold: reference strains. Trees are midpoint‐rooted.

**Figure S5.** Phylogenetic relationships of the HA gene of influenza viruses isolated in South Tyrol as obtained for A(H1N1)pdm09 using the neighbor‐joining method with a maximum composite likelihood substitution model; red and pink: vaccine strains; blue: strains from Italy; green: strains from Austria; bold: reference strains. Trees are midpoint‐rooted.

**Figure S6.** Phylogenetic relationships of the HA gene of influenza viruses isolated in South Tyrol as obtained for B (Victoria) using the neighbor‐joining method with a maximum composite likelihood substitution model; red and pink: vaccine strains; blue: strains from Italy; green: strains from Austria; bold: reference strains. Trees are midpoint‐rooted.

## Data Availability

The original data presented in the study are openly available in the online database GISAID at https://gisaid.org/. Metadata related to the sequences produced in this study are reported in Table S2.
