# Supplementary material for: Tracking Seasonal Influenza Trends in South Tyrol During 2022/2023 Using Genomic Surveillance Data
Source: Influenza Other Respir Viruses. 2025 Mar 26;19(4):e70083. doi: 10.1111/irv.70083 (PMC11946919; doi:10.1111/irv.70083)
Supplement: Supplementary file 1 — Table S1. Estimated mean and 95% confidence intervals for the reproduction number from genomic surveillance, length of infections, sequence sampling proportion, and time to most recent common ancestor according to different prior choices for the molecular clock and tree topology. Figure S7. Sequential steps of the analyses. The number of samples at each step is reported in the boxes. [file IRV-19-e70083-s009.docx]

Tracking seasonal influenza trends in South Tyrol during 2022-2023 using genomic surveillance data

**Supplementary Text**

**Birth-Death Skyline Model (BDSKY)**

The birth-death skyline model was implemented in the BEAST2 software (v2.7.5) using the BDSKY plugin.

Here, we report our prior assumptions in the parametrization of the BDSKY model using the same notation as in the BDSKY plugin for serial sampling (*ie* heterochronous data).

- The “becomeUninfectious” parameter is the rate at which infected patients become uninfectious, through recovery, death or isolation: becomeUninfectious ~ Lognormal(135,0.2).
- The “clockRate” parameter is the conversion rate between branch lengths and evolutionary time, by assuming a strict clock we assumed that every branch in the phylogenetic tree evolves according to the same evolutionary rate: clockRate ~ Lognormal(0.002,1.25).
- The ”origin_BDSKY_Serial” parameter is the duration of the epidemic represented by the samples under consideration: origin_BDSKY_Serial ~ Gamma(3,0.5).
- The “reproductionNumber“ parameter is the effective reproduction number, which denotes the average number of secondary infections caused by an infected person at a given time during the epidemic under consideration: reproductionNumber ~ Gamma(10,0.1).
- The “samplingProportion” parameter is the proportion of all removed lineages that were sampled and included in the analysis: samplingProportion ~ Lognormal(0.1,1).

Other parameter priors left to their default values

**Table S1**. Estimated mean and 95% confidence intervals for the reproduction number from genomic surveillance, length of infections, sequence sampling proportion and time to most recent common ancestor according to different prior choices for the molecular clock and tree topology.

| ***Influenza subtype*** | ***Scenario*** | ***Peak Re from phylodynamic*** | ***Duration of infectious period (days)*** | ***Sampling proportion*** | ***tMRCA (2022)*** |
| --- | --- | --- | --- | --- | --- |
| B(Victoria) | Baseline | 1.15  (1.02-1.29) | 4.07  (2.8-5.71) | 0.39%  (0.12-0.91%) | 20 Aug  (13 Jun - 10 Oct) |
| B(Victoria) | Optimized molecular clock | 1.15  (0.93-1.39) | 4.08  (2.8-5.7) | 0.35%  (0.09-0.91%) | 03 Sep  (23 Jun - 10 Nov) |
| B(Victoria) | Fixed tree topology | 1.15  (1.01-1.3) | 4.09  (2.81-5.75) | 0.4%  (0.12-0.96%) | 20 Aug  (15 Jun - 11 Oct) |
| A(H3N2) | Baseline | 1.24  (1.16-1.35) | 4.05  (2.89-5.48) | 0.23%  (0.09-0.47%) | 19 Feb  (28 Jan - 02 Mar) |
| A(H3N2) | Optimized molecular clock | 1.24  (1.16-1.35) | 4  (2.84-5.46) | 0.2%  (0.07-0.44%) | 17 Feb  (26 Jan - 02 Mar) |
| A(H3N2) | Fixed tree topology | 1.24  (1.16-1.34) | 4.04  (2.84-5.53) | 0.23%  (0.09-0.49%) | 19 Feb  (28 Jan - 02 Mar) |
| A(H1N1)pdm09 | Baseline | 1.19  (1.06-1.34) | 4.07  (2.79-5.76) | 0.39%  (0.11-0.98%) | 26 Mar  (05 Feb - 11 May) |
| A(H1N1)pdm09 | Optimized molecular clock | 1.27  (1.1-1.48) | 3.53  (2.45-4.91) | 0.93%  (0.26-2.28%) | 25 Sep  (09 Sep - 06 Oct) |
| A(H1N1)pdm09 | Fixed tree topology | 1.19  (1.06-1.35) | 4.09  (2.78-5.79) | 0.4%  (0.11-1.03%) | 26 Mar  (08 Feb - 10 May) |

**Supplementary Figure 7. Sequential steps of the analyses.** The number of samples at each step is reported in the boxes.

**
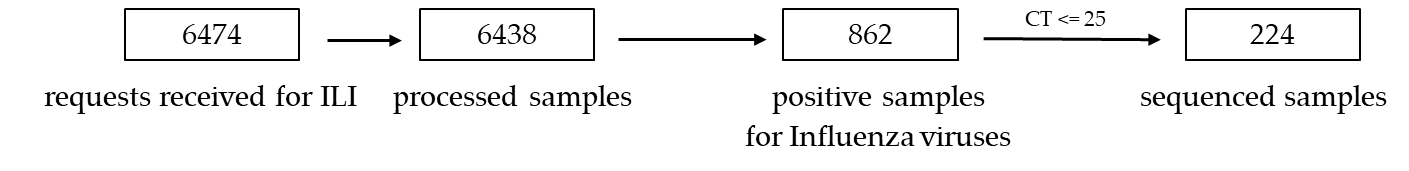
**
