# Supplementary material for: Tracking Seasonal Influenza Trends in South Tyrol During 2022/2023 Using Genomic Surveillance Data
Source: Influenza Other Respir Viruses. 2025 Mar 26;19(4):e70083. doi: 10.1111/irv.70083 (PMC11946919; doi:10.1111/irv.70083)
Supplement: Supplementary file 2 — Table S2. Metadata related to the isolates of the influenza viruses analyzed. [file IRV-19-e70083-s001.docx]

Tracking seasonal influenza trends in South Tyrol during 2022-2023 using genomic surveillance data

**Supplementary Table 2**. Metadata related to the isolates of the influenza viruses analysed.

| **Isolate name** | **ID GISAID** | **Isoltion date** | **Healthcare District** | **Patient age (years)** | **Influenza virus subtype** |
| --- | --- | --- | --- | --- | --- |
| A/Bolzano/173/2022 | EPI_ISL_18075991 | 01/09/2022 | Bressanone/Brixen | 70-79 | A / H3N2 |
| A/Bolzano/175/2022 | EPI_ISL_18075993 | 14/10/2022 | Bolzano/Bozen | 0-9 | A / H1N1 |
| A/Bolzano/174/2022 | EPI_ISL_18075992 | 14/10/2022 | Bressanone/Brixen | 80-89 | A / H3N2 |
| A/Bolzano/176/2022 | EPI_ISL_18075994 | 25/10/2022 | Brunico/Bruneck | 70-79 | A / H3N2 |
| A/Bolzano/177/2022 | EPI_ISL_18075995 | 28/10/2022 | Brunico/Bruneck | 10-19 | A / H3N2 |
| A/Bolzano/159/2022 | EPI_ISL_17464574 | 16/11/2022 | Merano/Meran | 40-49 | A / H3N2 |
| A/Bolzano/167/2022 | EPI_ISL_18075227 | 19/11/2022 | Bolzano/Bozen | 0-9 | A / H3N2 |
| A/Bolzano/155/2022 | EPI_ISL_17464570 | 22/11/2022 | Merano/Meran | 0-9 | A / H3N2 |
| A/Bolzano/158/2022 | EPI_ISL_17464573 | 22/11/2022 | Bolzano/Bozen | 0-9 | A / H3N2 |
| A/Bolzano/76/2022 | EPI_ISL_17454483 | 22/11/2022 | Bolzano/Bozen | 10-19 | A / H3N2 |
| A/Bolzano/157/2022 | EPI_ISL_17464572 | 22/11/2022 | Bolzano/Bozen | 10-19 | A / H3N2 |
| A/Bolzano/156/2022 | EPI_ISL_17464571 | 22/11/2022 | Bolzano/Bozen | 80-89 | A / H3N2 |
| A/Bolzano/77/2022 | EPI_ISL_17454484 | 25/11/2022 | Brunico/Bruneck | 10-19 | A / H3N2 |
| A/Bolzano/153/2022 | EPI_ISL_17464568 | 25/11/2022 | Bressanone/Brixen | 50-59 | A / H3N2 |
| A/Bolzano/154/2022 | EPI_ISL_17464569 | 25/11/2022 | Bolzano/Bozen | 80-89 | A / H3N2 |
| A/Bolzano/178/2022 | EPI_ISL_18075996 | 28/11/2022 | Bolzano/Bozen | 50-59 | A / H3N2 |
| A/Bolzano/161/2022 | EPI_ISL_18075221 | 28/11/2022 | Brunico/Bruneck | 80-89 | A / H3N2 |
| A/Bolzano/179/2022 | EPI_ISL_18075997 | 29/11/2022 | Brunico/Bruneck | 0-9 | A / H3N2 |
| A/Bolzano/180/2022 | EPI_ISL_18075998 | 30/11/2022 | Brunico/Bruneck | 0-9 | A / H3N2 |
| A/Bolzano/181/2022 | EPI_ISL_18075999 | 30/11/2022 | Bressanone/Brixen | 50-59 | A / H3N2 |
| A/Bolzano/168/2022 | EPI_ISL_18075228 | 30/11/2022 | Brunico/Bruneck | 80-89 | A / H3N2 |
| A/Bolzano/79/2022 | EPI_ISL_17454486 | 01/12/2022 | Bolzano/Bozen | 0-9 | A / H3N2 |
| A/Bolzano/80/2022 | EPI_ISL_17454487 | 01/12/2022 | Brunico/Bruneck | 0-9 | A / H3N2 |
| A/Bolzano/78/2022 | EPI_ISL_17454485 | 01/12/2022 | Brunico/Bruneck | 30-39 | A / H3N2 |
| A/Bolzano/169/2022 | EPI_ISL_18075229 | 01/12/2022 | Bolzano/Bozen | 30-39 | A / H3N2 |
| A/Bolzano/160/2022 | EPI_ISL_17464732 | 01/12/2022 | Bolzano/Bozen | 40-49 | A / H3N2 |
| A/Bolzano/151/2022 | EPI_ISL_17464592 | 01/12/2022 | Merano/Meran | 50-59 | A / H3N2 |
| A/Bolzano/152/2022 | EPI_ISL_17464567 | 01/12/2022 | Brunico/Bruneck | 70-79 | A / H3N2 |
| A/Bolzano/149/2022 | EPI_ISL_17464565 | 01/12/2022 | Bolzano/Bozen | 90-99 | A / H3N2 |
| A/Bolzano/182/2022 | EPI_ISL_18076000 | 02/12/2022 | Bolzano/Bozen | 0-9 | A / H1N1 |
| A/Bolzano/183/2022 | EPI_ISL_18076001 | 02/12/2022 | Bolzano/Bozen | 0-9 | A / H1N1 |
| A/Bolzano/162/2022 | EPI_ISL_18075222 | 02/12/2022 | Merano/Meran | 60-69 | A / H3N2 |
| A/Bolzano/163/2022 | EPI_ISL_18075223 | 02/12/2022 | Bolzano/Bozen | 70-79 | A / H3N2 |
| A/Bolzano/185/2022 | EPI_ISL_18076003 | 05/12/2022 | Brunico/Bruneck | 0-9 | A / H3N2 |
| A/Bolzano/184/2022 | EPI_ISL_18076002 | 05/12/2022 | Brunico/Bruneck | 0-9 | A / H3N2 |
| A/Bolzano/187/2022 | EPI_ISL_18076005 | 05/12/2022 | Brunico/Bruneck | 10-19 | A / H3N2 |
| A/Bolzano/150/2022 | EPI_ISL_17464566 | 05/12/2022 | Bolzano/Bozen | 60-69 | A / H3N2 |
| A/Bolzano/186/2022 | EPI_ISL_18076004 | 05/12/2022 | Bressanone/Brixen | 80-89 | A / H3N2 |
| A/Bolzano/85/2022 | EPI_ISL_17454492 | 06/12/2022 | Brunico/Bruneck | 10-19 | A / H3N2 |
| A/Bolzano/82/2022 | EPI_ISL_17454489 | 06/12/2022 | Bolzano/Bozen | 20-29 | A / H3N2 |
| A/Bolzano/83/2022 | EPI_ISL_17454490 | 06/12/2022 | Merano/Meran | 20-29 | A / H3N2 |
| A/Bolzano/102/2022 | EPI_ISL_17454509 | 06/12/2022 | Bolzano/Bozen | 80-89 | A / H3N2 |
| A/Bolzano/84/2022 | EPI_ISL_17454491 | 06/12/2022 | Merano/Meran | 90-99 | A / H3N2 |
| A/Bolzano/170/2022 | EPI_ISL_18075230 | 07/12/2022 | Brunico/Bruneck | 0-9 | A / H3N2 |
| A/Bolzano/107/2022 | EPI_ISL_17454514 | 07/12/2022 | Bressanone/Brixen | 0-9 | A / H3N2 |
| A/Bolzano/104/2022 | EPI_ISL_17454511 | 07/12/2022 | Brunico/Bruneck | 0-9 | A / H3N2 |
| A/Bolzano/109/2022 | EPI_ISL_17454516 | 07/12/2022 | Bressanone/Brixen | 40-49 | A / H3N2 |
| A/Bolzano/108/2022 | EPI_ISL_17454515 | 07/12/2022 | Bressanone/Brixen | 70-79 | A / H3N2 |
| A/Bolzano/105/2022 | EPI_ISL_17454512 | 07/12/2022 | Bolzano/Bozen | 70-79 | A / H3N2 |
| A/Bolzano/190/2022 | EPI_ISL_18076033 | 09/12/2022 | Bolzano/Bozen | 0-9 | A / H3N2 |
| A/Bolzano/164/2022 | EPI_ISL_18075224 | 09/12/2022 | Brunico/Bruneck | 0-9 | A / H3N2 |
| A/Bolzano/171/2022 | EPI_ISL_18075231 | 09/12/2022 | Bolzano/Bozen | 50-59 | A / H3N2 |
| A/Bolzano/189/2022 | EPI_ISL_18076032 | 09/12/2022 | Bolzano/Bozen | 80-89 | A / H3N2 |
| A/Bolzano/66/2022 | EPI_ISL_17072669 | 10/12/2022 | Bolzano/Bozen | 0-9 | A / H1N1 |
| A/Bolzano/113/2022 | EPI_ISL_17454520 | 10/12/2022 | Brunico/Bruneck | 0-9 | A / H3N2 |
| A/Bolzano/110/2022 | EPI_ISL_17454517 | 10/12/2022 | Brunico/Bruneck | 0-9 | A / H3N2 |
| A/Bolzano/117/2022 | EPI_ISL_17454524 | 10/12/2022 | Bressanone/Brixen | 0-9 | A / H3N2 |
| A/Bolzano/191/2022 | EPI_ISL_18076034 | 10/12/2022 | Brunico/Bruneck | 0-9 | A / H3N2 |
| A/Bolzano/115/2022 | EPI_ISL_17454522 | 10/12/2022 | Merano/Meran | 0-9 | A / H3N2 |
| A/Bolzano/119/2022 | EPI_ISL_17454526 | 10/12/2022 | Bolzano/Bozen | 0-9 | A / H3N2 |
| A/Bolzano/116/2022 | EPI_ISL_17454523 | 10/12/2022 | Merano/Meran | 10-19 | A / H3N2 |
| A/Bolzano/103/2022 | EPI_ISL_17454510 | 10/12/2022 | Bressanone/Brixen | 70-79 | A / H3N2 |
| A/Bolzano/114/2022 | EPI_ISL_17454521 | 10/12/2022 | Bolzano/Bozen | 70-79 | A / H3N2 |
| A/Bolzano/106/2022 | EPI_ISL_17454513 | 10/12/2022 | Brunico/Bruneck | 70-79 | A / H3N2 |
| A/Bolzano/111/2022 | EPI_ISL_17454518 | 10/12/2022 | Bolzano/Bozen | 80-89 | A / H3N2 |
| A/Bolzano/112/2022 | EPI_ISL_17454519 | 10/12/2022 | Brunico/Bruneck | 80-89 | A / H3N2 |
| A/Bolzano/118/2022 | EPI_ISL_17454525 | 10/12/2022 | Merano/Meran | 80-89 | A / H3N2 |
| A/Bolzano/192/2022 | EPI_ISL_18076035 | 11/12/2022 | Brunico/Bruneck | 0-9 | A / H3N2 |
| A/Bolzano/193/2022 | EPI_ISL_18076036 | 12/12/2022 | Bolzano/Bozen | 0-9 | A / H3N2 |
| A/Bolzano/194/2022 | EPI_ISL_18076110 | 12/12/2022 | Bressanone/Brixen | 20-29 | A / H3N2 |
| A/Bolzano/195/2022 | EPI_ISL_18076111 | 13/12/2022 | Bolzano/Bozen | 0-9 | A / H3N2 |
| A/Bolzano/172/2022 | EPI_ISL_18075990 | 14/12/2022 | Bolzano/Bozen | 0-9 | A / H3N2 |
| A/Bolzano/197/2022 | EPI_ISL_18076113 | 14/12/2022 | Bolzano/Bozen | 0-9 | A / H3N2 |
| A/Bolzano/196/2022 | EPI_ISL_18076112 | 14/12/2022 | Bolzano/Bozen | 50-59 | A / H1N1 |
| A/Bolzano/86/2022 | EPI_ISL_17454493 | 14/12/2022 | Merano/Meran | 90-99 | A / H3N2 |
| A/Bolzano/81/2022 | EPI_ISL_17454488 | 15/12/2022 | Bolzano/Bozen | 0-9 | A / H3N2 |
| A/Bolzano/166/2022 | EPI_ISL_18075226 | 16/12/2022 | Merano/Meran | 0-9 | A / H3N2 |
| A/Bolzano/165/2022 | EPI_ISL_18075225 | 16/12/2022 | Brunico/Bruneck | 0-9 | A / H3N2 |
| A/Bolzano/92/2022 | EPI_ISL_17454499 | 20/12/2022 | Bressanone/Brixen | 80-89 | A / H3N2 |
| A/Bolzano/54/2022 | EPI_ISL_17072637 | 21/12/2022 | Merano/Meran | 0-9 | A / H3N2 |
| A/Bolzano/55/2022 | EPI_ISL_17072638 | 21/12/2022 | Merano/Meran | 0-9 | A / H3N2 |
| A/Bolzano/91/2022 | EPI_ISL_17454498 | 21/12/2022 | Bressanone/Brixen | 10-19 | A / H3N2 |
| A/Bolzano/90/2022 | EPI_ISL_17454497 | 21/12/2022 | Bressanone/Brixen | 30-39 | A / H3N2 |
| A/Bolzano/67/2022 | EPI_ISL_17072649 | 21/12/2022 | Bressanone/Brixen | 40-49 | A / H1N1 |
| A/Bolzano/88/2022 | EPI_ISL_17454495 | 21/12/2022 | Bressanone/Brixen | 60-69 | A / H3N2 |
| A/Bolzano/52/2022 | EPI_ISL_17072635 | 21/12/2022 | Brunico/Bruneck | 70-79 | A / H3N2 |
| A/Bolzano/56/2022 | EPI_ISL_17072639 | 21/12/2022 | Merano/Meran | 80-89 | A / H3N2 |
| A/Bolzano/89/2022 | EPI_ISL_17454496 | 21/12/2022 | Bressanone/Brixen | 80-89 | A / H3N2 |
| A/Bolzano/57/2022 | EPI_ISL_17072640 | 21/12/2022 | Bolzano/Bozen | 80-89 | A / H3N2 |
| A/Bolzano/53/2022 | EPI_ISL_17072636 | 21/12/2022 | Brunico/Bruneck | 90-99 | A / H3N2 |
| A/Bolzano/87/2022 | EPI_ISL_17454494 | 21/12/2022 | Bressanone/Brixen | 90-99 | A / H3N2 |
| A/Bolzano/58/2022 | EPI_ISL_17072641 | 22/12/2022 | Bolzano/Bozen | 0-9 | A / H3N2 |
| A/Bolzano/101/2022 | EPI_ISL_17454508 | 22/12/2022 | Merano/Meran | 30-39 | A / H3N2 |
| A/Bolzano/94/2022 | EPI_ISL_17454501 | 23/12/2022 | Brunico/Bruneck | 0-9 | A / H3N2 |
| A/Bolzano/99/2022 | EPI_ISL_17454506 | 23/12/2022 | Merano/Meran | 0-9 | A / H3N2 |
| A/Bolzano/98/2022 | EPI_ISL_17454505 | 23/12/2022 | Merano/Meran | 0-9 | A / H3N2 |
| A/Bolzano/50/2022 | EPI_ISL_16997194 | 23/12/2022 | Brunico/Bruneck | 0-9 | A / H1N1 |
| A/Bolzano/100/2022 | EPI_ISL_17454507 | 23/12/2022 | Bolzano/Bozen | 30-39 | A / H3N2 |
| A/Bolzano/95/2022 | EPI_ISL_17454502 | 23/12/2022 | Brunico/Bruneck | 30-39 | A / H3N2 |
| A/Bolzano/51/2022 | EPI_ISL_16997292 | 23/12/2022 | Bolzano/Bozen | 50-59 | A / H1N1 |
| A/Bolzano/68/2022 | EPI_ISL_17072650 | 23/12/2022 | Brunico/Bruneck | 60-69 | A / H1N1 |
| A/Bolzano/96/2022 | EPI_ISL_17454503 | 23/12/2022 | Merano/Meran | 70-79 | A / H3N2 |
| A/Bolzano/93/2022 | EPI_ISL_17454500 | 23/12/2022 | Merano/Meran | 80-89 | A / H3N2 |
| A/Bolzano/97/2022 | EPI_ISL_17454504 | 23/12/2022 | Merano/Meran | 80-89 | A / H3N2 |
| A/Bolzano/59/2022 | EPI_ISL_17072642 | 24/12/2022 | Bolzano/Bozen | 0-9 | A / H3N2 |
| A/Bolzano/124/2022 | EPI_ISL_17454531 | 27/12/2022 | Brunico/Bruneck | 0-9 | A / H3N2 |
| A/Bolzano/60/2022 | EPI_ISL_17072643 | 27/12/2022 | Brunico/Bruneck | 0-9 | A / H3N2 |
| A/Bolzano/125/2022 | EPI_ISL_17454532 | 27/12/2022 | Brunico/Bruneck | 0-9 | A / H3N2 |
| A/Bolzano/142/2022 | EPI_ISL_17454556 | 27/12/2022 | Brunico/Bruneck | 0-9 | A / H3N2 |
| A/Bolzano/61/2022 | EPI_ISL_17072644 | 27/12/2022 | Brunico/Bruneck | 0-9 | A / H3N2 |
| A/Bolzano/121/2022 | EPI_ISL_17454528 | 27/12/2022 | Bolzano/Bozen | 0-9 | A / H3N2 |
| A/Bolzano/120/2022 | EPI_ISL_17454527 | 27/12/2022 | Brunico/Bruneck | 20-29 | A / H3N2 |
| A/Bolzano/122/2022 | EPI_ISL_17454529 | 27/12/2022 | Merano/Meran | 40-49 | A / H3N2 |
| A/Bolzano/123/2022 | EPI_ISL_17454530 | 27/12/2022 | Merano/Meran | 90-99 | A / H3N2 |
| A/Bolzano/69/2022 | EPI_ISL_17072670 | 28/12/2022 | Brunico/Bruneck | 0-9 | A / H1N1 |
| A/Bolzano/129/2022 | EPI_ISL_17454536 | 28/12/2022 | Merano/Meran | 0-9 | A / H3N2 |
| A/Bolzano/146/2022 | EPI_ISL_17454560 | 28/12/2022 | Brunico/Bruneck | 0-9 | A / H3N2 |
| A/Bolzano/63/2022 | EPI_ISL_17072646 | 28/12/2022 | Brunico/Bruneck | 0-9 | A / H3N2 |
| A/Bolzano/73/2022 | EPI_ISL_17072653 | 28/12/2022 | Bressanone/Brixen | 0-9 | A / H1N1 |
| A/Bolzano/72/2022 | EPI_ISL_17072671 | 28/12/2022 | Brunico/Bruneck | 0-9 | A / H1N1 |
| A/Bolzano/132/2022 | EPI_ISL_17454539 | 28/12/2022 | Merano/Meran | 0-9 | A / H3N2 |
| A/Bolzano/147/2022 | EPI_ISL_17454561 | 28/12/2022 | Brunico/Bruneck | 0-9 | A / H3N2 |
| A/Bolzano/134/2022 | EPI_ISL_17454541 | 28/12/2022 | Bressanone/Brixen | 0-9 | A / H3N2 |
| A/Bolzano/126/2022 | EPI_ISL_17454533 | 28/12/2022 | Brunico/Bruneck | 0-9 | A / H3N2 |
| A/Bolzano/128/2022 | EPI_ISL_17454535 | 28/12/2022 | Merano/Meran | 40-49 | A / H3N2 |
| A/Bolzano/135/2022 | EPI_ISL_17454542 | 28/12/2022 | Bressanone/Brixen | 40-49 | A / H3N2 |
| A/Bolzano/70/2022 | EPI_ISL_17072651 | 28/12/2022 | Brunico/Bruneck | 60-69 | A / H1N1 |
| A/Bolzano/143/2022 | EPI_ISL_17454557 | 28/12/2022 | Brunico/Bruneck | 70-79 | A / H3N2 |
| A/Bolzano/133/2022 | EPI_ISL_17454540 | 28/12/2022 | Merano/Meran | 70-79 | A / H3N2 |
| A/Bolzano/136/2022 | EPI_ISL_17454543 | 28/12/2022 | Merano/Meran | 70-79 | A / H3N2 |
| A/Bolzano/130/2022 | EPI_ISL_17454537 | 28/12/2022 | Merano/Meran | 70-79 | A / H3N2 |
| A/Bolzano/71/2022 | EPI_ISL_17072652 | 28/12/2022 | Brunico/Bruneck | 70-79 | A / H1N1 |
| A/Bolzano/144/2022 | EPI_ISL_17454558 | 28/12/2022 | Brunico/Bruneck | 70-79 | A / H3N2 |
| A/Bolzano/64/2022 | EPI_ISL_17072647 | 28/12/2022 | Brunico/Bruneck | 70-79 | A / H3N2 |
| A/Bolzano/131/2022 | EPI_ISL_17454538 | 28/12/2022 | Merano/Meran | 70-79 | A / H3N2 |
| A/Bolzano/148/2022 | EPI_ISL_17454562 | 28/12/2022 | Bressanone/Brixen | 70-79 | A / H3N2 |
| A/Bolzano/127/2022 | EPI_ISL_17454534 | 28/12/2022 | Merano/Meran | 80-89 | A / H3N2 |
| A/Bolzano/62/2022 | EPI_ISL_17072645 | 28/12/2022 | Brunico/Bruneck | 80-89 | A / H3N2 |
| A/Bolzano/145/2022 | EPI_ISL_17454559 | 28/12/2022 | Brunico/Bruneck | 80-89 | A / H3N2 |
| A/Bolzano/140/2022 | EPI_ISL_17454546 | 28/12/2022 | Bressanone/Brixen | 90-99 | A / H3N2 |
| A/Bolzano/74/2022 | EPI_ISL_17072654 | 29/12/2022 | Brunico/Bruneck | 0-9 | A / H1N1 |
| A/Bolzano/65/2022 | EPI_ISL_17072648 | 29/12/2022 | Bolzano/Bozen | 0-9 | A / H3N2 |
| A/Bolzano/138/2022 | EPI_ISL_17454545 | 29/12/2022 | Brunico/Bruneck | 60-69 | A / H3N2 |
| A/Bolzano/75/2022 | EPI_ISL_17072655 | 29/12/2022 | Merano/Meran | 70-79 | A / H1N1 |
| A/Bolzano/137/2022 | EPI_ISL_17454544 | 29/12/2022 | Merano/Meran | 80-89 | A / H3N2 |
| A/Bolzano/141/2022 | EPI_ISL_17454547 | 29/12/2022 | Merano/Meran | 80-89 | A / H3N2 |
| A/Bolzano/24/2023 | EPI_ISL_17454578 | 03/01/2023 | Bolzano/Bozen | 60-69 | A / H3N2 |
| A/Bolzano/26/2023 | EPI_ISL_17454580 | 03/01/2023 | Merano/Meran | 70-79 | A / H3N2 |
| A/Bolzano/30/2023 | EPI_ISL_17454576 | 03/01/2023 | Brunico/Bruneck | 80-89 | A / H3N2 |
| A/Bolzano/5/2023 | EPI_ISL_17072659 | 04/01/2023 | Brunico/Bruneck | 0-9 | A / H1N1 |
| A/Bolzano/4/2023 | EPI_ISL_17072672 | 04/01/2023 | Brunico/Bruneck | 0-9 | A / H1N1 |
| A/Bolzano/27/2023 | EPI_ISL_17454581 | 04/01/2023 | Bolzano/Bozen | 0-9 | A / H3N2 |
| A/Bolzano/12/2023 | EPI_ISL_17454549 | 04/01/2023 | Bressanone/Brixen | 0-9 | A / H3N2 |
| A/Bolzano/13/2023 | EPI_ISL_17454550 | 04/01/2023 | Brunico/Bruneck | 0-9 | A / H3N2 |
| A/Bolzano/25/2023 | EPI_ISL_17454579 | 04/01/2023 | Brunico/Bruneck | 10-19 | A / H3N2 |
| A/Bolzano/28/2023 | EPI_ISL_17454582 | 04/01/2023 | Merano/Meran | 70-79 | A / H3N2 |
| A/Bolzano/11/2023 | EPI_ISL_17454548 | 04/01/2023 | Bressanone/Brixen | 70-79 | A / H3N2 |
| A/Bolzano/23/2023 | EPI_ISL_17454577 | 04/01/2023 | Brunico/Bruneck | 80-89 | A / H3N2 |
| A/Bolzano/14/2023 | EPI_ISL_17454551 | 06/01/2023 | Merano/Meran | 0-9 | A / H3N2 |
| A/Bolzano/6/2023 | EPI_ISL_17072660 | 06/01/2023 | Brunico/Bruneck | 0-9 | A / H1N1 |
| A/Bolzano/15/2023 | EPI_ISL_17454552 | 07/01/2023 | Bressanone/Brixen | 0-9 | A / H3N2 |
| B/Bolzano/3/2023 | EPI_ISL_17072662 | 09/01/2023 | Brunico/Bruneck | 10-19 | B / Victoria |
| A/Bolzano/17/2023 | EPI_ISL_17454554 | 10/01/2023 | Brunico/Bruneck | 0-9 | A / H3N2 |
| A/Bolzano/18/2023 | EPI_ISL_17454555 | 10/01/2023 | Bressanone/Brixen | 60-69 | A / H3N2 |
| A/Bolzano/16/2023 | EPI_ISL_17454553 | 10/01/2023 | Brunico/Bruneck | 90-99 | A / H3N2 |
| A/Bolzano/19/2023 | EPI_ISL_17454563 | 12/01/2023 | Brunico/Bruneck | 0-9 | A / H3N2 |
| A/Bolzano/33/2023 | EPI_ISL_18075232 | 12/01/2023 | Merano/Meran | 30-39 | A / H3N2 |
| A/Bolzano/7/2023 | EPI_ISL_17072661 | 16/01/2023 | Bolzano/Bozen | 0-9 | A / H1N1 |
| A/Bolzano/20/2023 | EPI_ISL_17454564 | 16/01/2023 | Brunico/Bruneck | 0-9 | A / H3N2 |
| B/Bolzano/7/2023 | EPI_ISL_17072666 | 17/01/2023 | Merano/Meran | 0-9 | B / Victoria |
| B/Bolzano/4/2023 | EPI_ISL_17072663 | 17/01/2023 | Brunico/Bruneck | 10-19 | B / Victoria |
| B/Bolzano/5/2023 | EPI_ISL_17072664 | 17/01/2023 | Bressanone/Brixen | 30-39 | B / Victoria |
| B/Bolzano/6/2023 | EPI_ISL_17072665 | 17/01/2023 | Merano/Meran | 60-69 | B / Victoria |
| A/Bolzano/21/2023 | EPI_ISL_17454565 | 17/01/2023 | Merano/Meran | 70-79 | A / H3N2 |
| A/Bolzano/22/2023 | EPI_ISL_17454566 | 18/01/2023 | Brunico/Bruneck | 0-9 | A / H3N2 |
| A/Bolzano/32/2023 | EPI_ISL_17465751 | 20/01/2023 | Brunico/Bruneck | 20-29 | A / H3N2 |
| B/Bolzano/9/2023 | EPI_ISL_17072673 | 25/01/2023 | Merano/Meran | 0-9 | B / Victoria |
| B/Bolzano/8/2023 | EPI_ISL_17072667 | 25/01/2023 | Merano/Meran | 30-39 | B / Victoria |
| A/Bolzano/1/2023 | EPI_ISL_17072656 | 25/01/2023 | Bressanone/Brixen | 70-79 | A / H3N2 |
| A/Bolzano/2/2023 | EPI_ISL_17072657 | 25/01/2023 | Bressanone/Brixen | 80-89 | A / H3N2 |
| A/Bolzano/3/2023 | EPI_ISL_17072658 | 25/01/2023 | Merano/Meran | 80-89 | A / H3N2 |
| B/Bolzano/10/2023 | EPI_ISL_17072668 | 26/01/2023 | Bolzano/Bozen | 20-29 | B / Victoria |
| B/Bolzano/28/2023 | EPI_ISL_17464564 | 30/01/2023 | Merano/Meran | 30-39 | B / Victoria |
| B/Bolzano/27/2023 | EPI_ISL_17464563 | 08/02/2023 | Merano/Meran | 20-29 | B / Victoria |
| A/Bolzano/31/2023 | EPI_ISL_17465749 | 08/02/2023 | Bressanone/Brixen | 50-59 | A / H1N1 |
| B/Bolzano/34/2023 | EPI_ISL_18075238 | 14/02/2023 | Merano/Meran | 10-19 | B / Victoria |
| B/Bolzano/12/2023 | EPI_ISL_17334009 | 15/02/2023 | Merano/Meran | 20-29 | B / Victoria |
| B/Bolzano/11/2023 | EPI_ISL_17334008 | 15/02/2023 | Merano/Meran | 50-59 | B / Victoria |
| B/Bolzano/14/2023 | EPI_ISL_17334011 | 17/02/2023 | Brunico/Bruneck | 0-9 | B / Victoria |
| B/Bolzano/13/2023 | EPI_ISL_17334010 | 17/02/2023 | Brunico/Bruneck | 10-19 | B / Victoria |
| B/Bolzano/29/2023 | EPI_ISL_18075233 | 18/02/2023 | Bolzano/Bozen | 0-9 | B / Victoria |
| B/Bolzano/16/2023 | EPI_ISL_17334013 | 21/02/2023 | Merano/Meran | 20-29 | B / Victoria |
| A/Bolzano/8/2023 | EPI_ISL_17334005 | 22/02/2023 | Bressanone/Brixen | 0-9 | A / H1N1 |
| B/Bolzano/17/2023 | EPI_ISL_17334014 | 22/02/2023 | Merano/Meran | 30-39 | B / Victoria |
| B/Bolzano/18/2023 | EPI_ISL_17334015 | 23/02/2023 | Brunico/Bruneck | 0-9 | B / Victoria |
| B/Bolzano/19/2023 | EPI_ISL_17334016 | 23/02/2023 | Merano/Meran | 10-19 | B / Victoria |
| B/Bolzano/20/2023 | EPI_ISL_17334017 | 24/02/2023 | Brunico/Bruneck | 10-19 | B / Victoria |
| B/Bolzano/22/2023 | EPI_ISL_17334019 | 24/02/2023 | Bolzano/Bozen | 20-29 | B / Victoria |
| B/Bolzano/30/2023 | EPI_ISL_18075234 | 27/02/2023 | Merano/Meran | 20-29 | B / Victoria |
| A/Bolzano/9/2023 | EPI_ISL_17334006 | 27/02/2023 | Bressanone/Brixen | 70-79 | A / H1N1 |
| A/Bolzano/10/2023 | EPI_ISL_17334007 | 27/02/2023 | Bressanone/Brixen | 80-89 | A / H1N1 |
| B/Bolzano/23/2023 | EPI_ISL_17334020 | 28/02/2023 | Brunico/Bruneck | 0-9 | B / Victoria |
| B/Bolzano/21/2023 | EPI_ISL_17334018 | 01/03/2023 | Bolzano/Bozen | 0-9 | B / Victoria |
| B/Bolzano/25/2023 | EPI_ISL_17334022 | 02/03/2023 | Merano/Meran | 50-59 | B / Victoria |
| B/Bolzano/24/2023 | EPI_ISL_17334021 | 03/03/2023 | Bolzano/Bozen | 0-9 | B / Victoria |
| B/Bolzano/26/2023 | EPI_ISL_17334023 | 07/03/2023 | Brunico/Bruneck | 20-29 | B / Victoria |
| A/Bolzano/35/2023 | EPI_ISL_18076218 | 10/03/2023 | Merano/Meran | 10-19 | A / H1N1 |
| B/Bolzano/37/2023 | EPI_ISL_18075241 | 10/03/2023 | Bressanone/Brixen | 20-29 | B / Victoria |
| A/Bolzano/36/2023 | EPI_ISL_18076219 | 14/03/2023 | Brunico/Bruneck | 0-9 | A / H3N2 |
| A/Bolzano/37/2023 | EPI_ISL_18076220 | 14/03/2023 | Bressanone/Brixen | 70-79 | A / H1N1 |
| B/Bolzano/41/2023 | EPI_ISL_18076221 | 16/03/2023 | Brunico/Bruneck | 0-9 | B / Victoria |
| B/Bolzano/42/2023 | EPI_ISL_18076222 | 17/03/2023 | Brunico/Bruneck | 0-9 | B / Victoria |
| B/Bolzano/43/2023 | EPI_ISL_18076223 | 17/03/2023 | Bolzano/Bozen | 10-19 | B / Victoria |
| B/Bolzano/38/2023 | EPI_ISL_18075242 | 20/03/2023 | Brunico/Bruneck | 0-9 | B / Victoria |
| B/Bolzano/44/2023 | EPI_ISL_18076224 | 20/03/2023 | Brunico/Bruneck | 30-39 | B / Victoria |
| B/Bolzano/15/2023 | EPI_ISL_17334012 | 21/03/2023 | Bressanone/Brixen | 0-9 | B / Victoria |
| B/Bolzano/45/2023 | EPI_ISL_18076225 | 21/03/2023 | Bressanone/Brixen | 30-39 | B / Victoria |
| B/Bolzano/46/2023 | EPI_ISL_18076226 | 22/03/2023 | Brunico/Bruneck | 0-9 | B / Victoria |
| A/Bolzano/38/2023 | EPI_ISL_18076227 | 22/03/2023 | Merano/Meran | 50-59 | A / H1N1 |
| A/Bolzano/39/2023 | EPI_ISL_18076228 | 23/03/2023 | Brunico/Bruneck | 50-59 | A / H1N1 |
| A/Bolzano/34/2023 | EPI_ISL_18075244 | 27/03/2023 | Brunico/Bruneck | 0-9 | A / H1N1 |
| B/Bolzano/39/2023 | EPI_ISL_18075243 | 27/03/2023 | Brunico/Bruneck | 10-19 | B / Victoria |
| B/Bolzano/32/2023 | EPI_ISL_18075236 | 11/04/2023 | Brunico/Bruneck | 0-9 | B / Victoria |
| B/Bolzano/33/2023 | EPI_ISL_18075237 | 11/04/2023 | Brunico/Bruneck | 0-9 | B / Victoria |
