# Supplementary material for: Tracking Seasonal Influenza Trends in South Tyrol During 2022/2023 Using Genomic Surveillance Data
Source: Influenza Other Respir Viruses. 2025 Mar 26;19(4):e70083. doi: 10.1111/irv.70083 (PMC11946919; doi:10.1111/irv.70083)
Supplement: Supplementary file 3 — Data S1. Supplementary information. [file IRV-19-e70083-s002.docx]

Tracking seasonal influenza trends in South Tyrol during 2022-2023 using genomic surveillance data

**Captions Supplementary Figures**

**Supplementary Figure 1**. Phylogenetic relationships of the HA gene of influenza viruses isolated in South Tyrol as obtained for A(H3N2) using the Maximal Likelihood (ML) method with a Tamura-Nei substitution; red and pink: vaccine strains; blue: representing strains from Italy; green: representing strains from Austria; bold: reference strains. Trees are midpoint-rooted.

**Supplementary Figure 2**. Phylogenetic relationships of the HA gene of influenza viruses isolated in South Tyrol as obtained for A(H1N1)pdm09 using the Maximal Likelihood (ML) method with a Tamura-Nei substitution; red and pink: vaccine strains; blue: representing strains from Italy; green: representing strains from Austria; bold: reference strains. Trees are midpoint-rooted.

**Supplementary Figure 3**. Phylogenetic relationships of the HA gene of influenza viruses isolated in South Tyrol as obtained for B(Victoria) using the Maximal Likelihood (ML) method with a Tamura-Nei substitution; red and pink: vaccine strains; blue: representing strains from Italy; green: representing strains from Austria; bold: reference strains. Trees are midpoint-rooted.

**Supplementary Figure 4**. Phylogenetic relationships of the HA gene of influenza viruses isolated in South Tyrol as obtained for A(H3N2) using the Neighbor-Joining method with a Maximum Composite Likelihood substitution model; red and pink: vaccine strains; blue: representing strains from Italy; green: representing strains from Austria; bold: reference strains. Trees are midpoint-rooted.

**Supplementary Figure 5**. Phylogenetic relationships of the HA gene of influenza viruses isolated in South Tyrol as obtained for A(H1N1)pdm09 using the Neighbor-Joining method with a Maximum Composite Likelihood substitution model; red and pink: vaccine strains; blue: representing strains from Italy; green: representing strains from Austria; bold: reference strains. Trees are midpoint-rooted.

**Supplementary Figure 6**. Phylogenetic relationships of the HA gene of influenza viruses isolated in South Tyrol as obtained for B(Victoria) using the Neighbor-Joining method with a Maximum Composite Likelihood substitution model; red and pink: vaccine strains; blue: representing strains from Italy; green: representing strains from Austria; bold: reference strains. Trees are midpoint-rooted.
