# Supplementary figures and images for: Tracking Seasonal Influenza Trends in South Tyrol During 2022/2023 Using Genomic Surveillance Data
Source: Influenza Other Respir Viruses. 2025 Mar 26;19(4):e70083. doi: 10.1111/irv.70083 (PMC11946919; doi:10.1111/irv.70083)

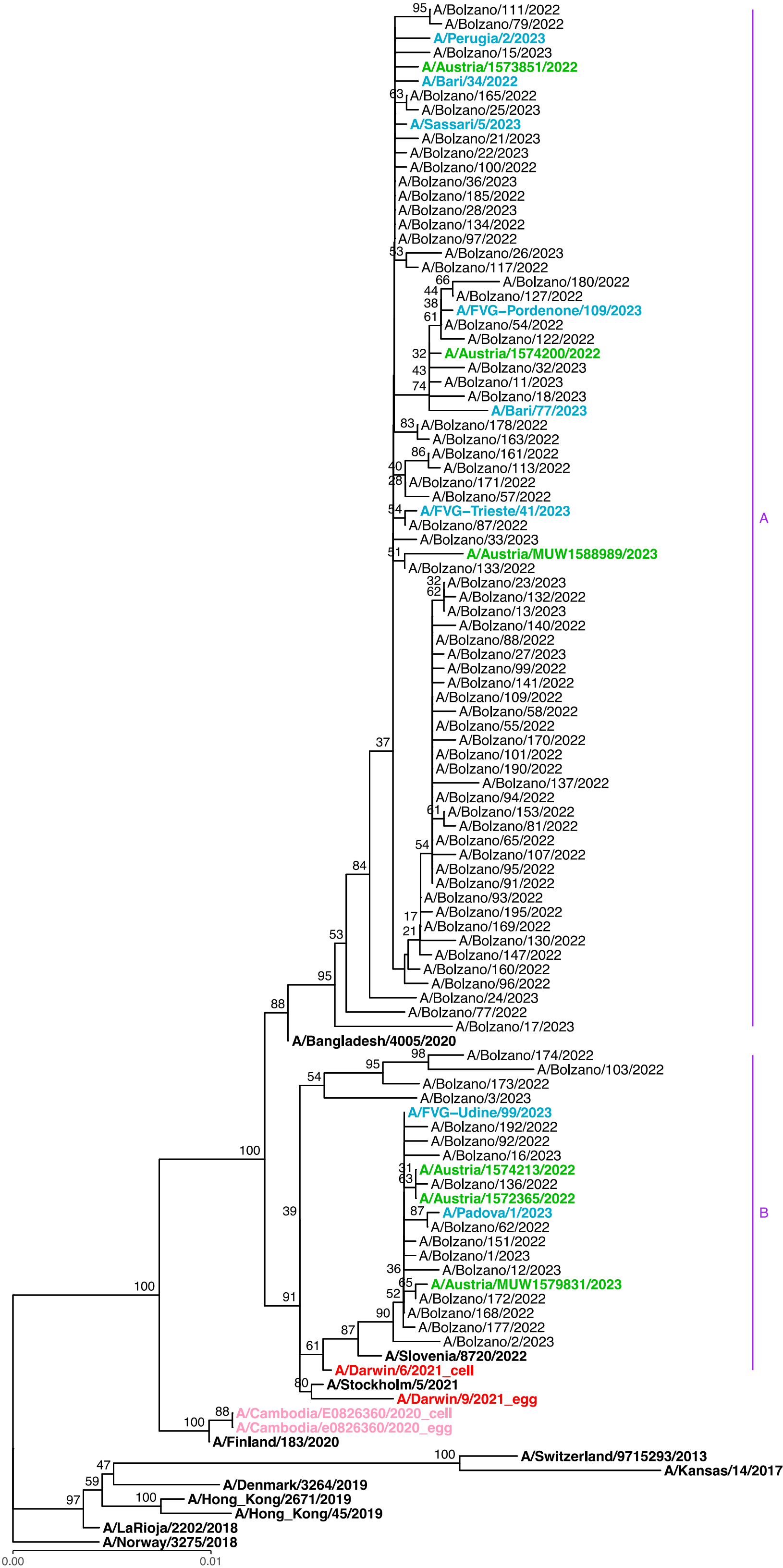

A

3C.2a1b.2a.2b

3C.2a1b.2a.2a.3a  
3C.2a1b.2a.2a.3a.1  
3C.2a1b.2a.2a.3  
3C.2a1b.2a.2a.1

B

3C.2a1b.2a.2a.1b

Supplement: Supplementary file 4 — Figure S1. Phylogenetic relationships of the HA gene of influenza viruses isolated in South Tyrol as obtained for A(H3N2) using the maximal likelihood (ML) method with a Tamura–Nei substitution; red and pink: vaccine strains; blue: strains from Italy; green: strains from Austria; bold: reference strains. Trees are midpoint‐rooted. [file IRV-19-e70083-s003.pdf]

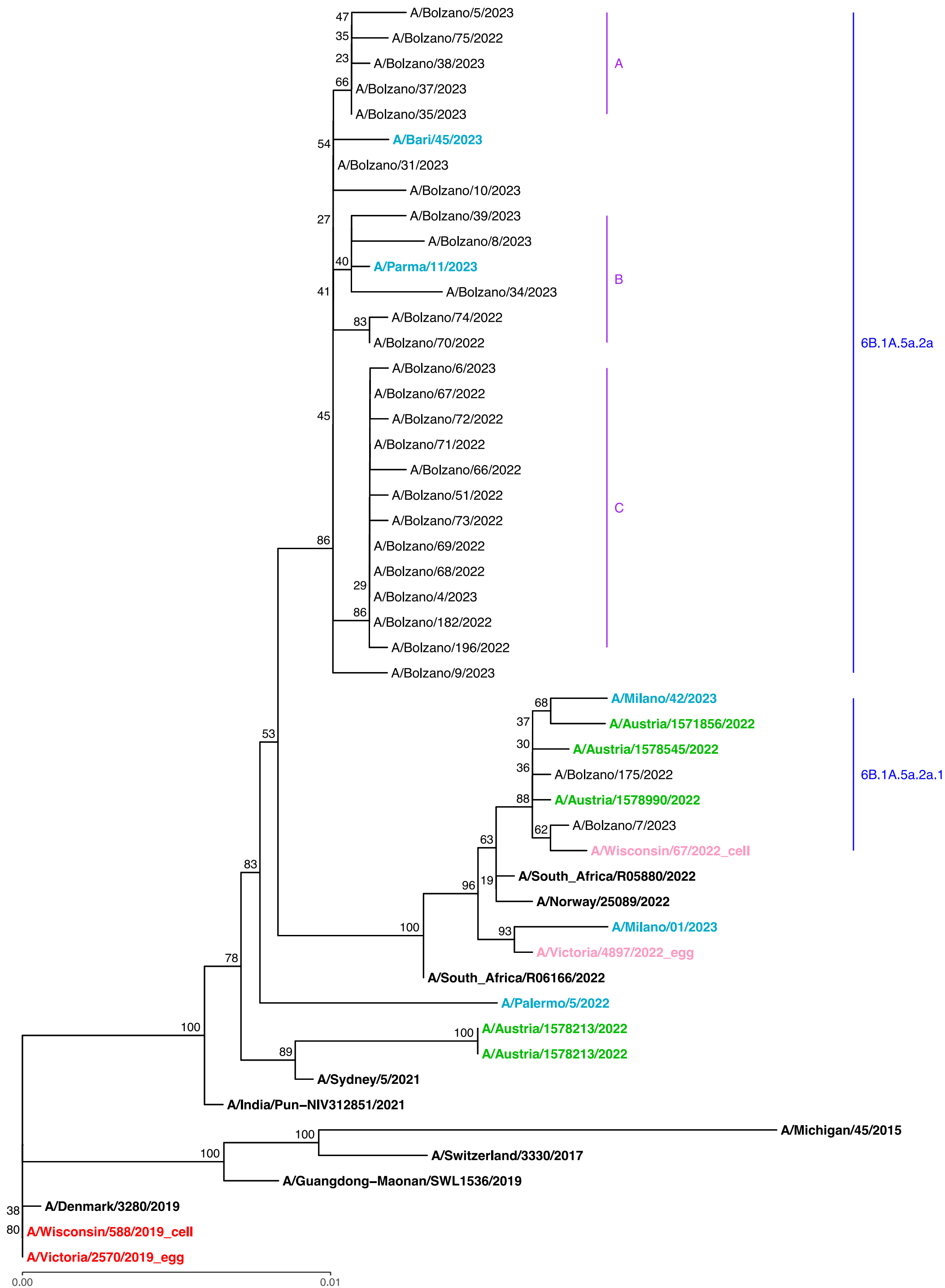

Supplement: Supplementary file 5 — Figure S2. Phylogenetic relationships of the HA gene of influenza viruses isolated in South Tyrol as obtained for A(H1N1)pdm09 using the maximal likelihood (ML) method with a Tamura–Nei substitution; red and pink: vaccine strains; blue: strains from Italy; green: strains from Austria; bold: reference strains. Trees are midpoint‐rooted. [file IRV-19-e70083-s005.pdf]

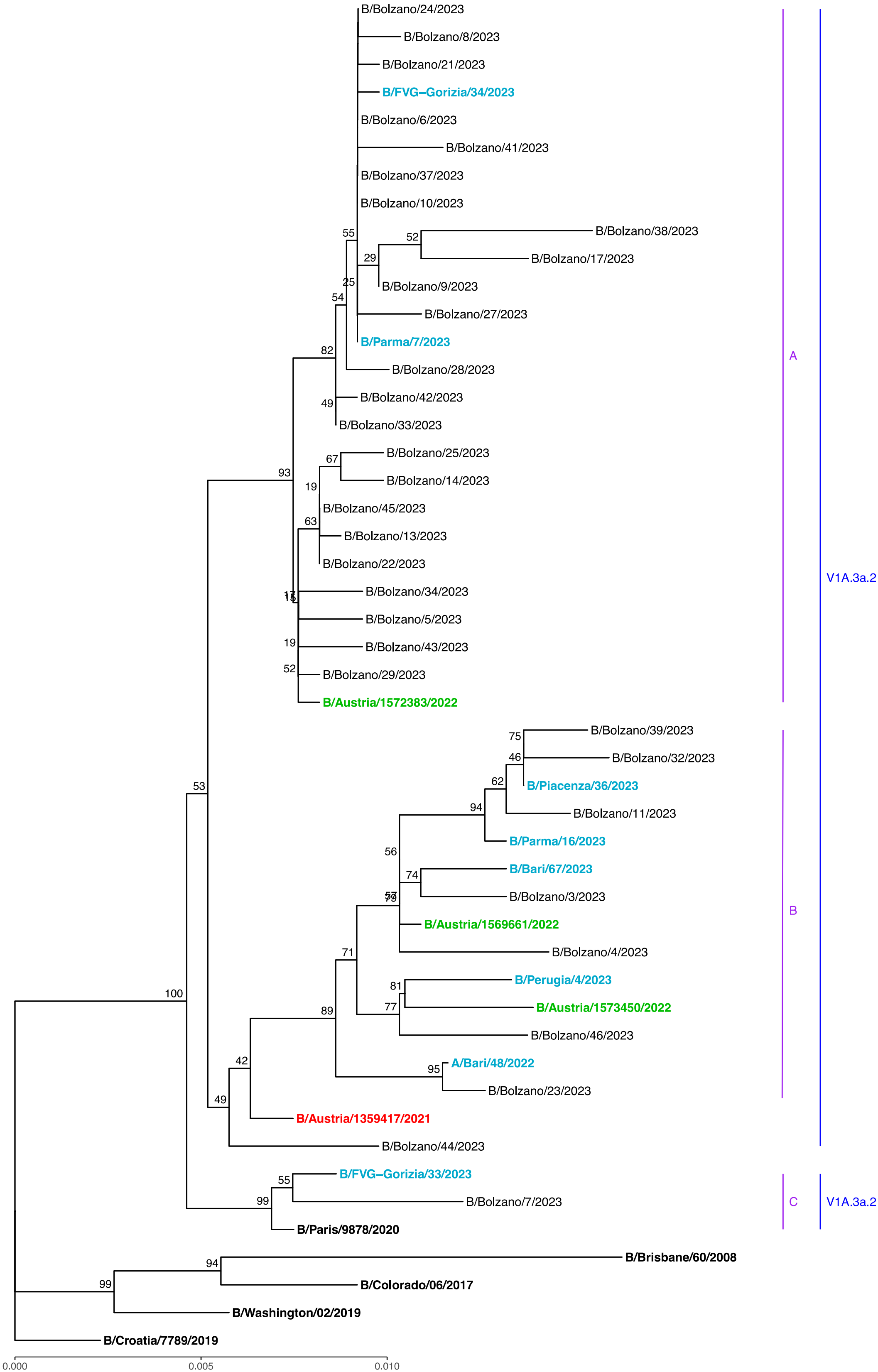

Supplement: Supplementary file 6 — Figure S3. Phylogenetic relationships of the HA gene of influenza viruses isolated in South Tyrol as obtained for B (Victoria) using the maximal likelihood (ML) method with a Tamura–Nei substitution; red and pink: vaccine strains; blue: strains from Italy; green: strains from Austria; bold: reference strains. Trees are midpoint‐rooted. [file IRV-19-e70083-s008.pdf]

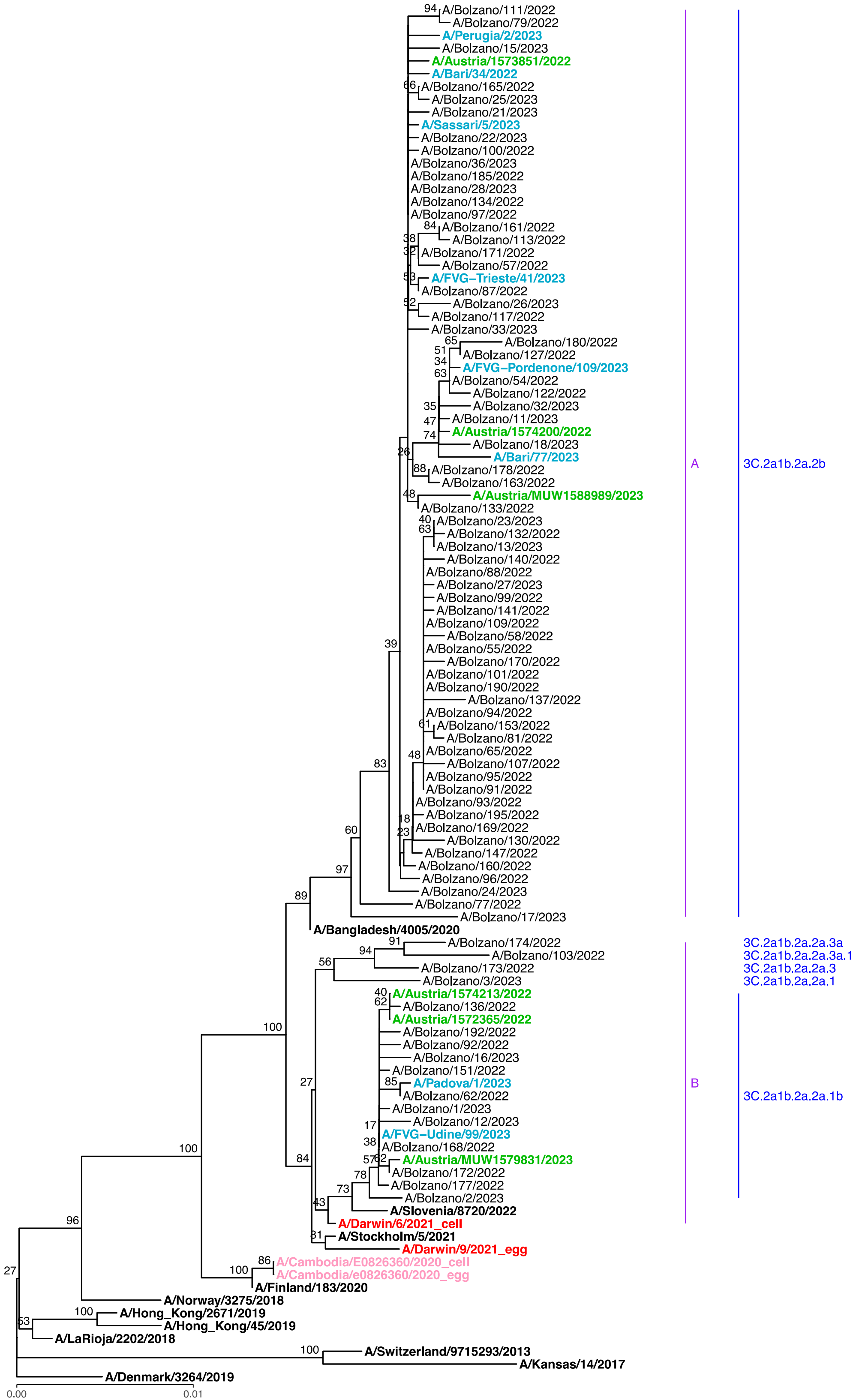

Supplement: Supplementary file 7 — Figure S4. Phylogenetic relationships of the HA gene of influenza viruses isolated in South Tyrol as obtained for A(H3N2) using the neighbor‐joining method with a maximum composite likelihood substitution model; red and pink: vaccine strains; blue: strains from Italy; green: strains from Austria; bold: reference strains. Trees are midpoint‐rooted. [file IRV-19-e70083-s006.pdf]

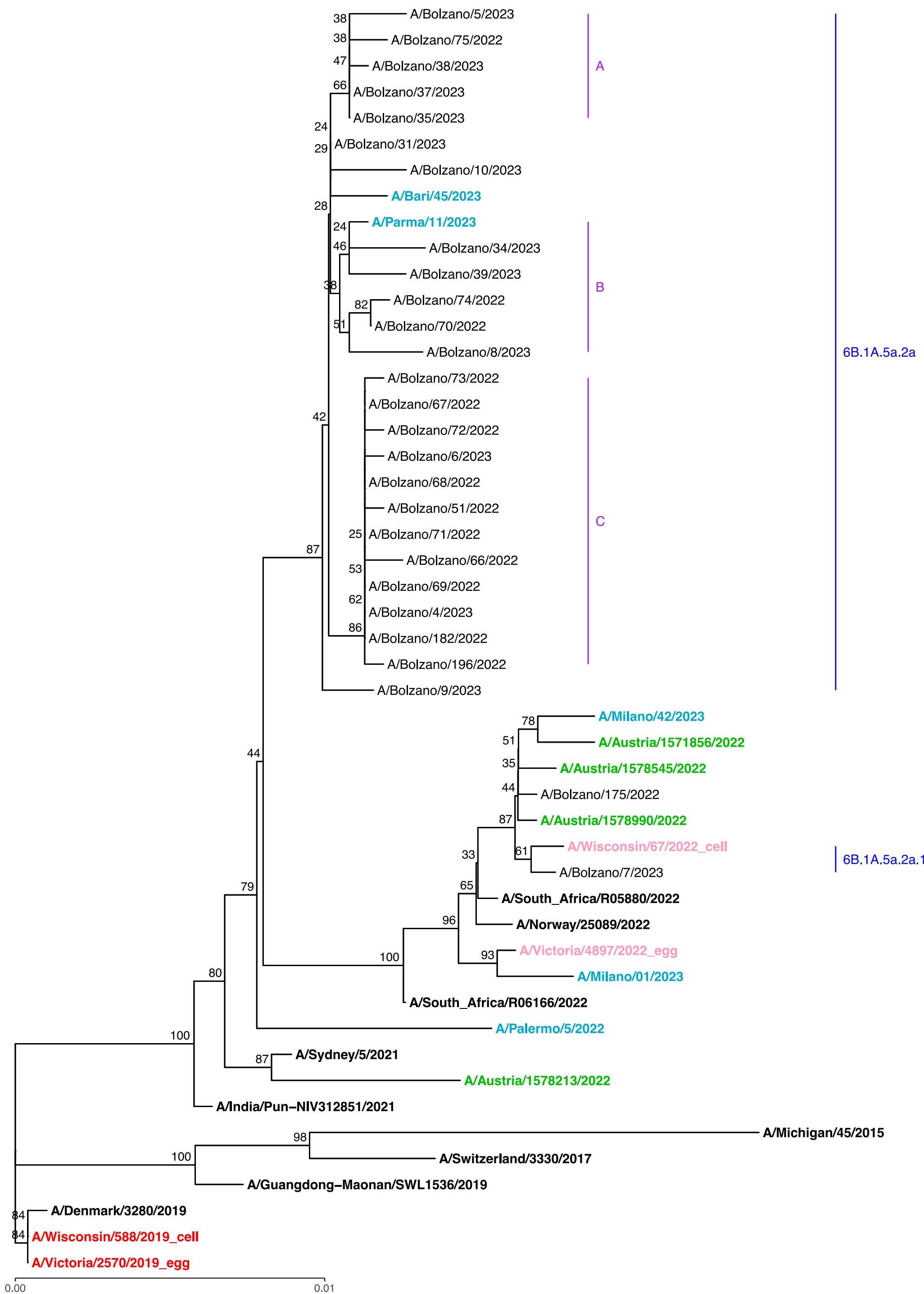

Supplement: Supplementary file 8 — Figure S5. Phylogenetic relationships of the HA gene of influenza viruses isolated in South Tyrol as obtained for A(H1N1)pdm09 using the neighbor‐joining method with a maximum composite likelihood substitution model; red and pink: vaccine strains; blue: strains from Italy; green: strains from Austria; bold: reference strains. Trees are midpoint‐rooted. [file IRV-19-e70083-s004.pdf]

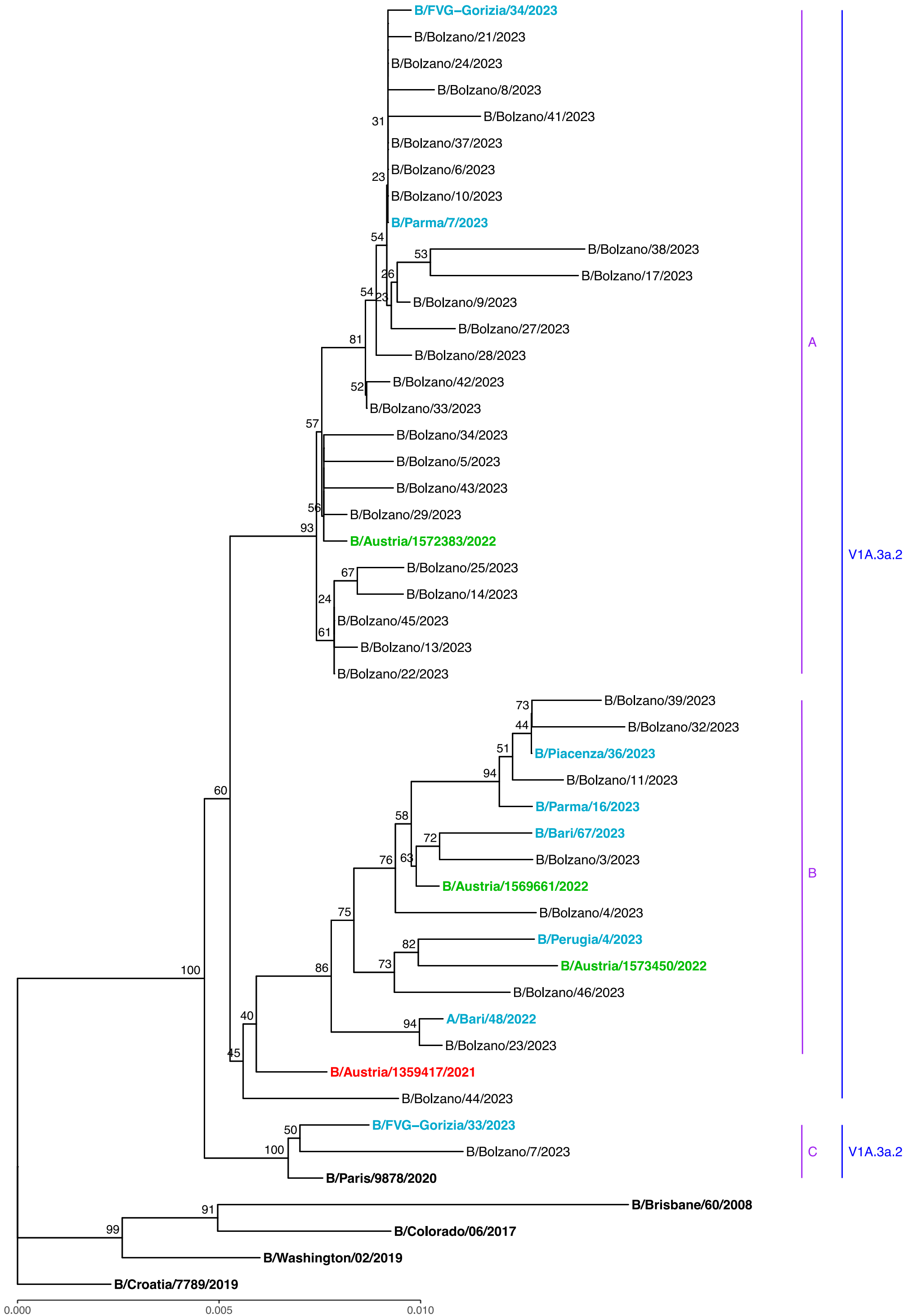

Supplement: Supplementary file 9 — Figure S6. Phylogenetic relationships of the HA gene of influenza viruses isolated in South Tyrol as obtained for B (Victoria) using the neighbor‐joining method with a maximum composite likelihood substitution model; red and pink: vaccine strains; blue: strains from Italy; green: strains from Austria; bold: reference strains. Trees are midpoint‐rooted. [file IRV-19-e70083-s007.pdf]
